# Global research landscape and trends of single-cell sequencing in sexually transmitted infections: a comprehensive bibliometric analysis from 2015 to 2025

**DOI:** 10.3389/frph.2026.1817416

**Published:** 2026-07-13

**Authors:** Cuixiu Wu, Yuanshuo Guo, Na Cui, Yidan Gao, Lijuan Jiang, Haoneng Tang, Qingchun Liang, Lingli Tang

**Affiliations:** 1 Department of Laboratory Medicine, The Second Xiangya Hospital, Central South University, Changsha, Hunan, China; 2 Department of Pathology, The Second Xiangya Hospital, Central South University, Changsha, Hunan, China; 3 Hunan Clinical Medical Research Center for Cancer Pathogenic Genes Testing and Diagnosis, Changsha, Hunan, China

**Keywords:** bibliometric analysis, human immunodeficiency virus (HIV), human papillomavirus (HPV), sexually transmitted infections, single-cell sequencing

## Abstract

**Background:**

Single-cell sequencing offers new insights into sexually transmitted infections (STIs) research, but there remains a lack of systematic bibliometric overview. Here, we summarize and quantify the developmental dynamics, research hotspots, and future directions of single-cell sequencing in STIs through bibliometric analysis.

**Methods:**

Relevant publications from 2015 to 2025 were retrieved from the Web of Science Core Collection (WoSCC), and data were visualized and analyzed using CiteSpace, VOSviewer, and the Bibliometrix R package. In addition, clinical trials were obtained from ClinicalTrials.gov to assess clinical advancements in this field. It should be noted that the data for 2025 were incomplete, as only publications indexed up to the search cutoff date (May 1, 2025) were included.

**Results:**

A total of 148 relevant articles and reviews were included in this study. The United States led in terms of publication output, citation counts, international collaboration, and influence. Chiarella J, Ho YC, and Wang FS were the most active authors in this field, while Stuart T was the most frequently co-cited scholar. Analysis of co-cited references identified “HPV-related head and neck squamous cell carcinoma” and “HIV-regulating factor” as clusters with significantly increased citation activity in recent years, suggesting emerging research attention. Keyword clustering objectively outlined the current research landscape, including studies on immune responses and pathogenic mechanisms of HIV infection, research on HIV latent reservoirs and viral persistence, investigations into HPV-related cancers, as well as single-cell and spatial transcriptomics-based immune mapping. Additionally, several emerging keywords were identified, including “spatial transcriptomics”, “epithelial cells”, “biomarkers”, and “machine learning”. Clinical trial analysis indicated both the promise and the ongoing challenges of clinical translation.

**Conclusions:**

By applying bibliometric methods to comprehensively summarize the research landscape and advances of single-cell sequencing in STIs, this study enhances the understanding of research hotspots and emerging directions in the field and provides references for future research.

## 1 Introduction

Sexually transmitted infections (STIs) comprise a heterogeneous group of infectious diseases caused by bacteria, viruses, and parasites, and are mainly transmitted through sexual contact, including vaginal, anal, and oral sex (1). STIs remain a major global public health concern, with their burden exacerbated by regional socioeconomic inequalities and uneven access to healthcare services. The World Health Organization (WHO) estimates that approximately 374 million new cases of curable STIs occur worldwide each year, highlighting the persistent pressure on health systems (2). Major STI pathogens—such as HIV, HPV, *Chlamydia trachomatis*, *Neisseria gonorrhoeae*, and *Treponema pallidum*—contribute not only to morbidity and mortality but also to long-term sequelae, including infertility, adverse pregnancy outcomes, malignancies, and enhanced HIV transmission (3–6). Despite advances in antimicrobials and vaccines, key challenges remain, including antimicrobial resistance (e.g., multidrug-resistant *N. gonorrhoeae*), persistent infection reservoirs (e.g., HIV latency), and incompletely understood mechanisms driving complications (7–9). Therefore, elucidating pathogen-host cellular interactions, site-specific immune responses, microenvironmental dynamics, and persistence/resistance mechanisms is essential for improving prevention, diagnosis, and treatment strategies.

Recent breakthroughs in single-cell sequencing have transformed infectious disease research by enabling high-resolution profiling of cellular genomes, epigenomes, transcriptomes, proteomes, and metabolomes at the individual-cell level (10). Compared with bulk population-based assays, single-cell sequencing resolves cellular heterogeneity, captures rare or transient cell states, and reduces population-averaging effects that may mask key signals (11). In STI research, single-cell sequencing is increasingly used to build immune atlases, dissect host–pathogen interaction networks, characterize immune repertoires, and prioritize candidate biomarkers or therapeutic targets (1). In HIV-1 studies, single-cell RNA sequencing (scRNA-seq) has generated mechanistic insights into latency-associated programs (12, 13), infection-associated inflammatory mechanisms (14), and immune landscape remodeling during chronic infection (15), and has facilitated the identification of functionally distinct immune subsets associated with viral control and immunopathogenesis (16, 17). Collectively, single-cell sequencing offers a powerful framework to connect cellular heterogeneity with functional dynamics and microenvironmental interactions in STIs, thereby supporting translational efforts from mechanistic discovery to clinically relevant biomarker development and therapeutic innovation.

With the rapid advancement of single-cell sequencing technologies in STIs, related research outputs have surged significantly. However, there is still a lack of systematic analysis on the distribution of research themes, knowledge evolution trajectory, and international collaboration landscape in this field. Bibliometrics, which employs mathematical operations and statistical methods to describe or reveal relationships within published works, serves as an invaluable tool for quantitatively assessing scientific publications and research trends (18). Compared with literature reviews, bibliometric analysis is more objective and reliable (19). Accordingly, we conducted a comprehensive bibliometric analysis of global single-cell sequencing research in STIs from 2015 to 2025 to: (1) map the knowledge landscape and identify key contributors and collaboration networks; (2) characterize thematic hotspots and track paradigm shifts; and (3) summarize challenges and emerging opportunities to inform future translational priorities.

## 2 Methods

### 2.1 Data collection

**Figure 1:**
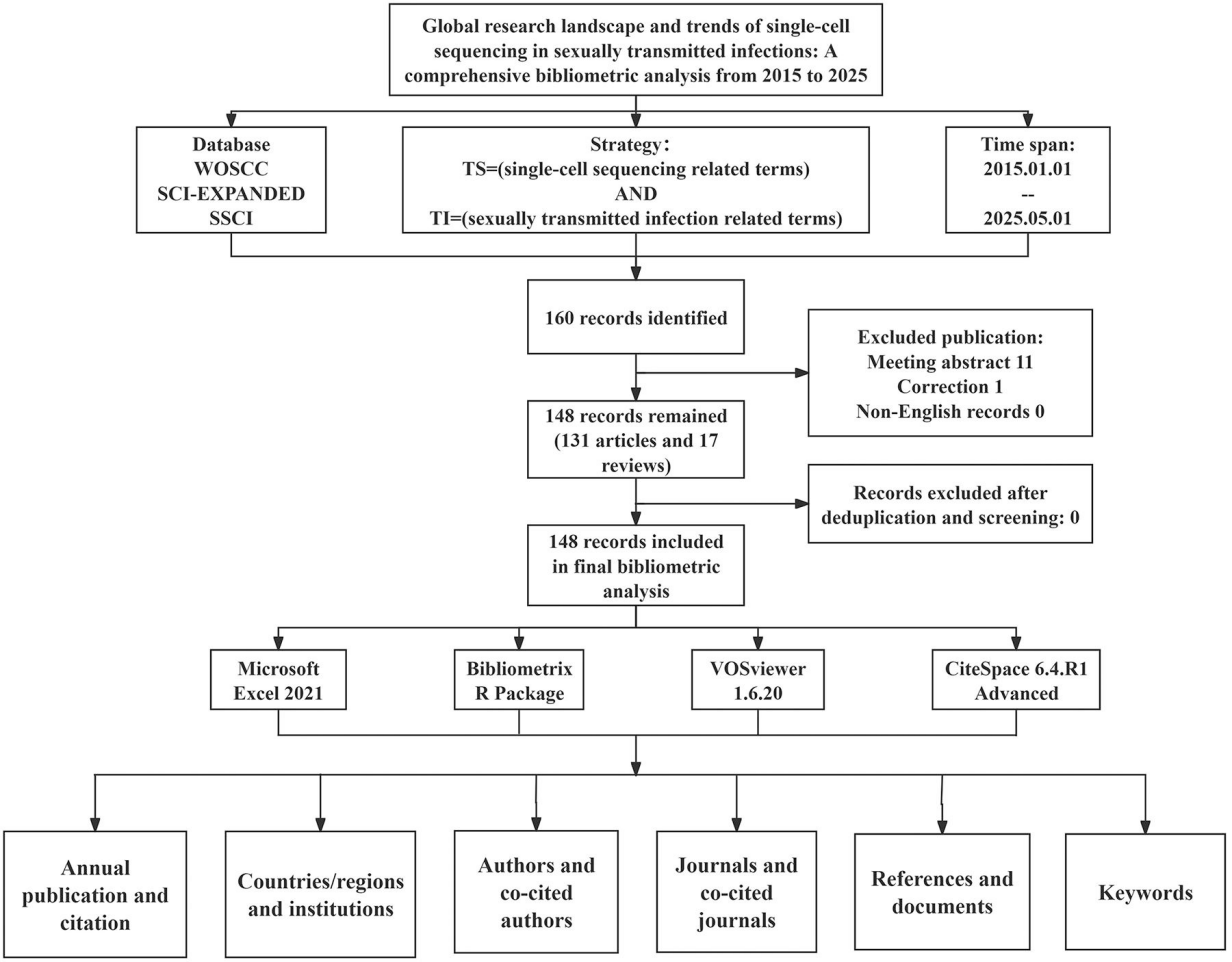
Flow chart of this study.

The Web of Science Core Collection (WoSCC) was selected as the primary data source for this bibliometric analysis due to its broad multidisciplinary coverage and its comprehensive metadata for citation analysis and collaboration network construction (20). Data were retrieved via Central South University's institutional subscription on May 22, 2025, and included only records from Science Citation Index Expanded (SCI-EXPANDED) and Social Sciences Citation Index (SSCI) databases. The specific search formula is as follows: TS = (“single-cell sequencing” OR “single-cell genom*” OR “single-cell DNA seq*” OR “scDNA-seq” OR “single-cell transcriptom*” OR “single-cell RNA seq*” OR “scRNA-seq” OR “single-cell metabolom*” OR “single-cell proteom*” OR “single-cell epigenom*” OR “scATAC-seq” OR “single-cell omics” OR “single-cell multiomics” OR “single-cell immune profiling”) AND TI = (“sexually transmitted infection*” OR “sexually transmitted disease*” OR “lymphogranuloma venereum” OR “chlamydia trachomatis” OR “genital chlamydia” OR “syphilis” OR “treponema pallidum” OR “HSV-2” OR “genital herpes” OR “genital ulcer” OR “human papillomavirus” OR “HPV” OR “human immunodeficiency virus” OR “HIV” OR “chancroid” OR “haemophilus ducreyi”) AND DOP = (2015-01-01/2025-05-01) AND DT = (Article OR Review) AND LA = (English). To validate the sensitivity of our title-restricted search strategy, we performed a comparative analysis using an alternative Topic-field search; the detailed results are provided in Supplementary Table S1. Based on this validation, we retained the original strategy for the main bibliometric analyses to ensure dataset homogeneity and minimize off-topic inclusions. Figure 1 illustrates the detailed screening process (TS, topics; T, title; DOP, publication date; DT, document type; LA, language).

We retrieved clinical trials from ClinicalTrials.gov for the period from January 1, 2015, to May 1, 2025, using the following search terms: (“single-cell sequencing” OR “single-cell genom*” OR “single-cell DNA seq*” OR “scDNA-seq” OR “single-cell transcriptom*” OR “single-cell RNA seq*” OR “scRNA-seq” OR “single-cell metabolom*” OR “single-cell proteom*” OR “single-cell epigenom*” OR “scATAC-seq” OR “single-cell omics” OR “single-cell multiomics” OR “single-cell immune profiling”) AND (“sexually transmitted infection*” OR “sexually transmitted disease*” OR “lymphogranuloma venereum” OR “chlamydia trachomatis” OR “genital chlamydia” OR “syphilis” OR “treponema pallidum” OR “HSV-2” OR “genital herpes” OR “genital ulcer” OR “human papillomavirus” OR “HPV” OR “human immunodeficiency virus” OR “HIV” OR “chancroid” OR “haemophilus ducreyi”). The applied filters included: study type (interventional and observational), study status (all), and age group (child, adult, and older adult). No restrictions were placed on study phase, location, or funding. Trials were included only if they explicitly applied single-cell sequencing technologies in STI-related research.

### 2.2 Data analysis

All literature and citation information were exported from the WoSCC database in .txt format. No duplicate records were found after automatic and manual checking using EndNote X9. Therefore, no deduplication was applied. Subsequently, this study employed various data visualization and knowledge mapping tools for the bibliometric analysis, including Microsoft Excel 2021, R software (version 4.4.2, http://www.bibliometrix.org), VOSviewer (version 1.6.20), and CiteSpace (version 6.4.R1 Advanced). Microsoft Excel 2021 was used to display annual publication counts and citation trends, and to handle the merging of keywords with different formats but identical or similar meanings (Supplementary Table S10). The Bibliometrix R Package is an open-source tool for scientometric analysis and visualization, co-developed by statisticians Massimo Aria and Corrado Cuccurullo (19). This package was used to extract publication counts, citation data, and international collaboration metrics across countries/regions, and these data were visualized using bar charts and chord diagrams. VOSviewer (Visualization of Similarities viewer) is a tool for constructing and visualizing bibliometric networks (21). We used VOSviewer to analyze and visualize author collaboration networks, journal/reference co-citation networks, and keyword co-occurrence networks. CiteSpace is an open-source Java-based tool developed by Professor Chaomei Chen from Drexel University for visualizing and analyzing scientific literature trends (22). Specifically, it can identify key milestones, intellectual turning points, and critical moments in the field's evolution (23). We applied CiteSpace to perform co-occurrence analyses of countries/regions and institutions, dual-map overlay analysis of journals, timeline analyses of keywords and references, and burst detection for institutions, references, and keywords. Additionally, nodes with betweenness centrality values ≥0.1 were marked with purple circles to highlight the importance of publications (or authors, journals, countries, etc.) in the field (24). Detailed parameter configurations for all visualization outputs are provided in the Supplementary Table S9 and Supplementary Figures S2–S21. It should be noted that all bibliometric analyses conducted herein are exploratory and descriptive in intent, and no corrections for multiple comparisons were applied. Results should be interpreted as visualizations and descriptive trends of the knowledge domain.

### 2.3 Validity and reliability

Two independent reviewers (Yuanshuo Guo and Na Cui) manually screened the records by title and abstract to assess relevance to the research topic. Any disagreements were resolved through discussion or consultation with a third researcher. To evaluate the robustness of our findings and to address potential database coverage bias, we additionally performed a validation analysis using the PubMed/MEDLINE database. The detailed search strategy are provided in the Supplementary Table S2.

## 3 Results

### 3.1 Publication and citation trend and pathogen distribution profile

**Figure 2:**
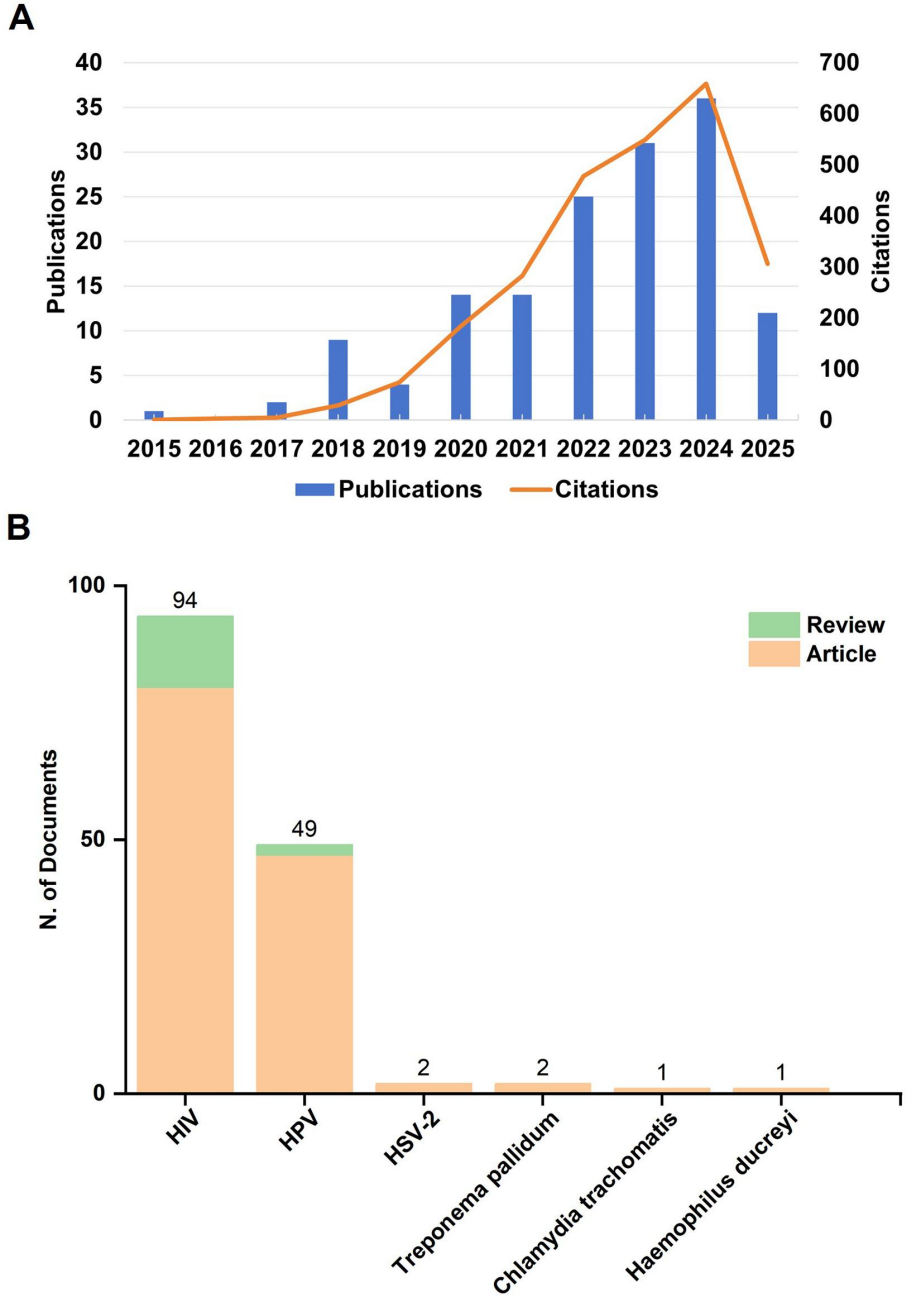
Publication and citation trend and pathogen distribution profile. **(A)** Annual publication and citation growth trends of research on single-cell sequencing in STIs from 2015 to 2025. **(B)** Distribution of included publications by STI pathogen type, divided into original articles and reviews. Note: data for 2025 were incomplete.

A total of 148 publications were retrieved from the WoSCC database using our predefined search strategy, comprising 131 articles and 17 reviews. These publications accumulated 2,360 non-self-citations, with an H-index of 27 and an average of 17.36 citations per publication. Annual publication volume and citation frequency are core indicators reflecting the development trends of this research field. As shown in Figure 2A, research applying single-cell sequencing to STIs has gained increasing traction and scholarly attention over the past decade. Based on annual publication output and citation metrics, the development of this field over the past decade can be divided into two distinct phases. The initial phase (2015–2019) was characterized by slow growth, with research emerging and scholars beginning to explore the field, yet yielding limited outputs and citation impact. The second phase (2020–2024) saw rapid and sustained development. Notably, the 2025 data only cover publications from January 1 to May 1, 2025 (the search cutoff date), and thus do not represent a full calendar year. The apparent decline in 2025 should be interpreted with caution, as it likely reflects the truncated time window rather than an actual decrease in research activity.

To clarify the research scope of included studies, we classified all publications according to their target STI pathogens (Figure 2B). Overall, studies on HIV (*n* = 94) and HPV (*n* = 49) accounted for the vast majority of included publications. Among them, HIV-related studies included 79 original articles and 15 reviews, and HPV-related studies included 47 original articles and 2 reviews. In contrast, research on other STI pathogens was extremely scarce. There were only 2 studies on HSV-2 and *Treponema pallidum* respectively, and only 1 study each on *Chlamydia trachomatis* and *Haemophilus ducreyi*. Notably, one publication focusing on HIV/HSV-2 co-infection was counted in both pathogen categories, resulting in the sum of subgroup counts (*n* = 149) being slightly higher than the total number of included publications (*n* = 148). These results indicated that current single-cell sequencing research in the STI field is highly concentrated on HIV and HPV.

### 3.2 Contribution of countries/regions and institutions

**Figure 3:**
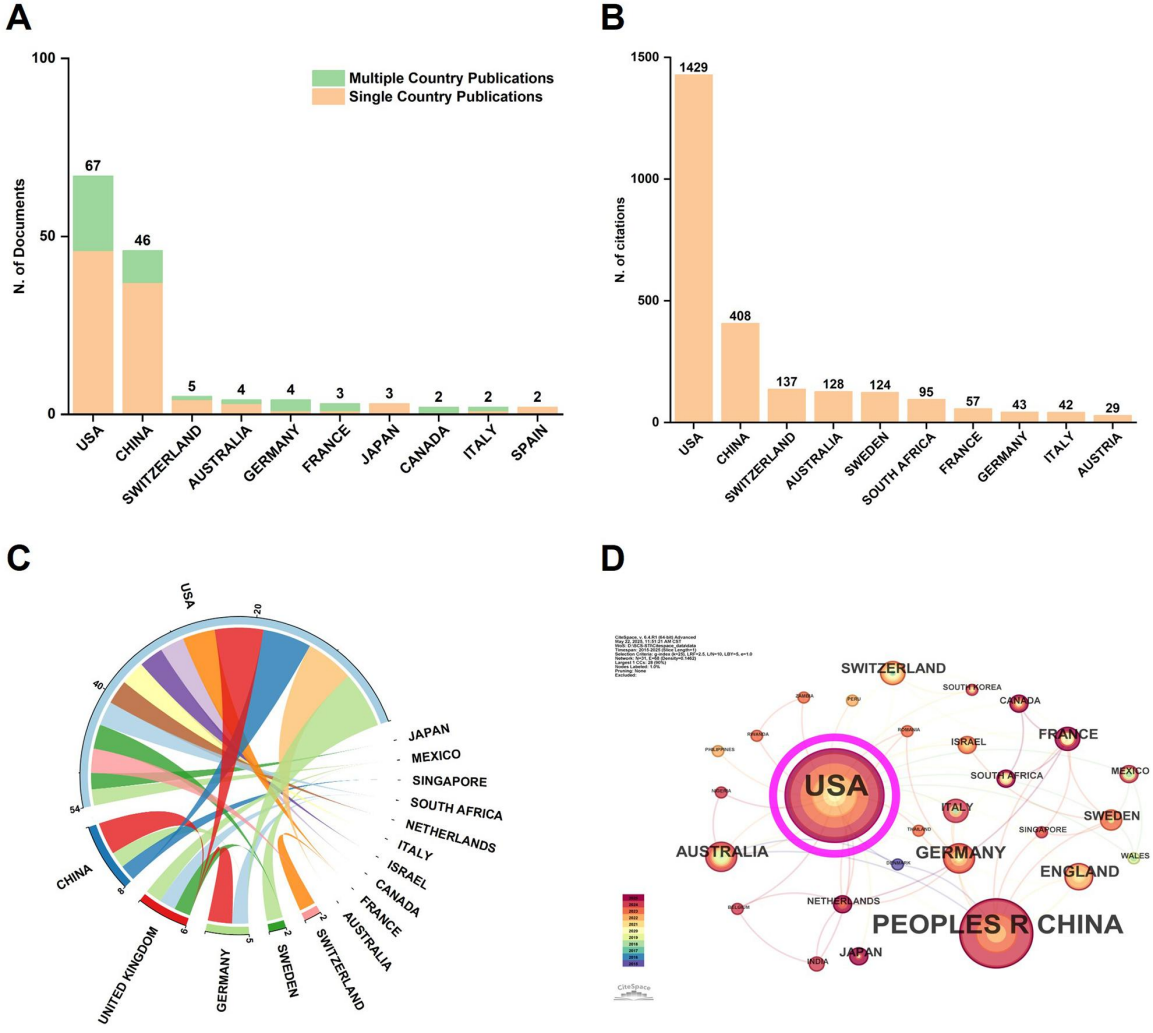
Contribution of different countries/regions to publications on this topic. **(A)** The top 10 corresponding authors’ countries. **(B)** The top 10 most cited countries/regions. **(C)** International cooperation analysis (cooperation frequency > 2). Lines between countries/regions represent cooperative relationships, with thicker lines indicating more frequent and closer cooperation. **(D)** The network map of countries/regions from CiteSpace. Node size represents the total number of publications for a country/region, with larger nodes indicating a greater number of publications. The purple outer ring around a node signifies high betweenness centrality, indicating its importance in the network.

Analysis of corresponding authors' countries using the Bibliometrix R Package (Figure 3A) revealed that the United States (*n* = 67, 45.3%) had the highest number of publications, followed by China (*n* = 46, 31.1%) and Switzerland (*n* = 5, 3.4%). The United States also frequently collaborated with other countries and regions, contributing to 21 international co-authored publications (31.3% of its total output). In terms of citation frequencies (Figure 3B), the United States had the highest number of citations (*n* = 1,429), followed by China (*n* = 408) and Switzerland (*n* = 137). Figure 3C illustrates the global international collaborative landscape, showing that the United States exhibited the strongest collaborative linkages, most notably with Germany, Australia, China, and the United Kingdom. For Figure 3D, CiteSpace was applied to map the international collaborative network. The United States occupied a pivotal position in this research field. Overall, countries should enhance international exchanges and scientific cooperation to foster synergistic innovation and advance scientific progress.

**Figure 4:**
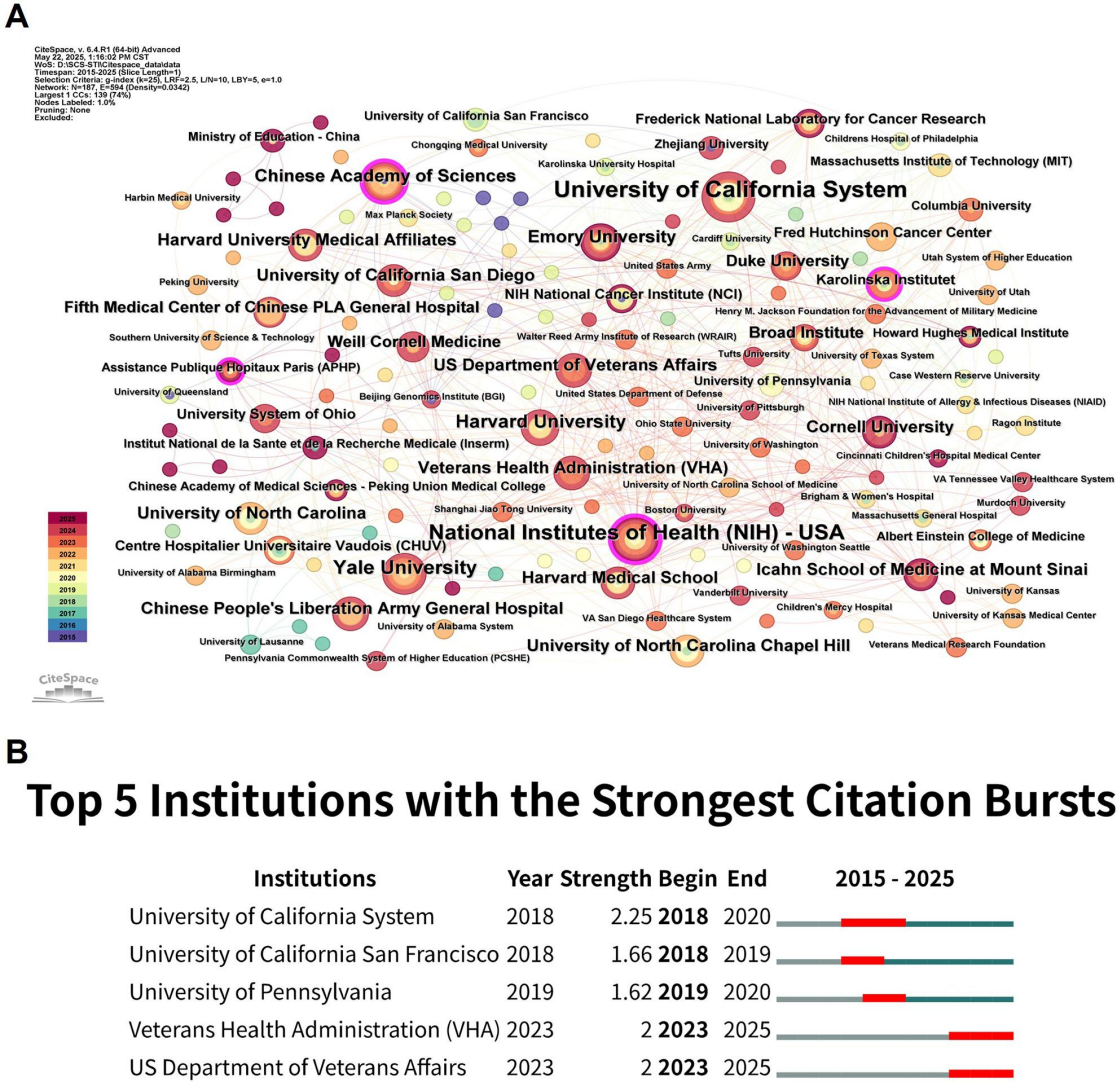
Contribution of different institutions to publications on this topic. **(A)** The network map of institutions from CiteSpace. Node size represents the total number of publications for an institution, with larger nodes indicating a greater number of publications. The purple outer ring around a node signifies high betweenness centrality, indicating its importance in the network. **(B)** Top 5 institutions with the strongest citation bursts from CiteSpace (*γ* = 0.6). Institutions marked with red bars indicate a sudden surge in citation frequency during the corresponding period.

Table 1 lists the top 10 institutions ranked by publication output and centrality, respectively. In terms of publication output, the University of California System ranked first with 14 publications, followed by the National Institutes of Health (NIH)—USA (*n* = 12), Yale University (*n* = 9), and Harvard University (*n* = 8). Of these 10 institutions, 9 are from the United States and 1 is from China. Centrality analysis shows that the Chinese Academy of Sciences (0.28) and the National Institutes of Health (NIH)—USA (0.27) ranked first and second, respectively. The co-occurrence network analysis of institutions based on CiteSpace (Figure 4A) shows that four institutions, including the Chinese Academy of Sciences, the National Institutes of Health (NIH)—USA, Assistance Publique Hopitaux Paris (APHP), and Karolinska Institutet, are marked with purple outer rings, highlighting their key role as bridges in the network. Figure 4B shows the top 5 institutions with the strongest citation bursts. Among them, the Veterans Health Administration (VHA) and the US Department of Veterans Affairs saw their citation bursts during 2023–2025, reflecting rising scholarly attention to their related studies in recent years.

**Table 1:** Top 10 institutions ranked by count and centrality.

Rank	Institution	Count	Rank	Institution	Centrality
1	University of California System	14	1	Chinese Academy of Sciences	0.28
2	National Institutes of Health (NIH)—USA	12	2	National Institutes of Health (NIH)—USA	0.27
3	Yale University	9	3	Assistance Publique Hopitaux Paris (APHP)	0.18
4	Harvard University	8	4	Karolinska Institutet	0.15
5	Chinese Academy of Sciences	7	5	Institut National de la Sante et de la Recherche Medicale (Inserm)	0.09
6	Emory University	7	6	Southern University of Science and Technology	0.09
7	Harvard Medical School	6	7	Emory University	0.08
8	Icahn School of Medicine at Mount Sinai	6	8	Broad Institute	0.08
9	University of North Carolina	6	9	Harbin Medical University	0.08
10	University of North Carolina Chapel Hill	6	10	Harvard Medical School	0.07

### 3.3 Analysis of authors

**Figure 5:**
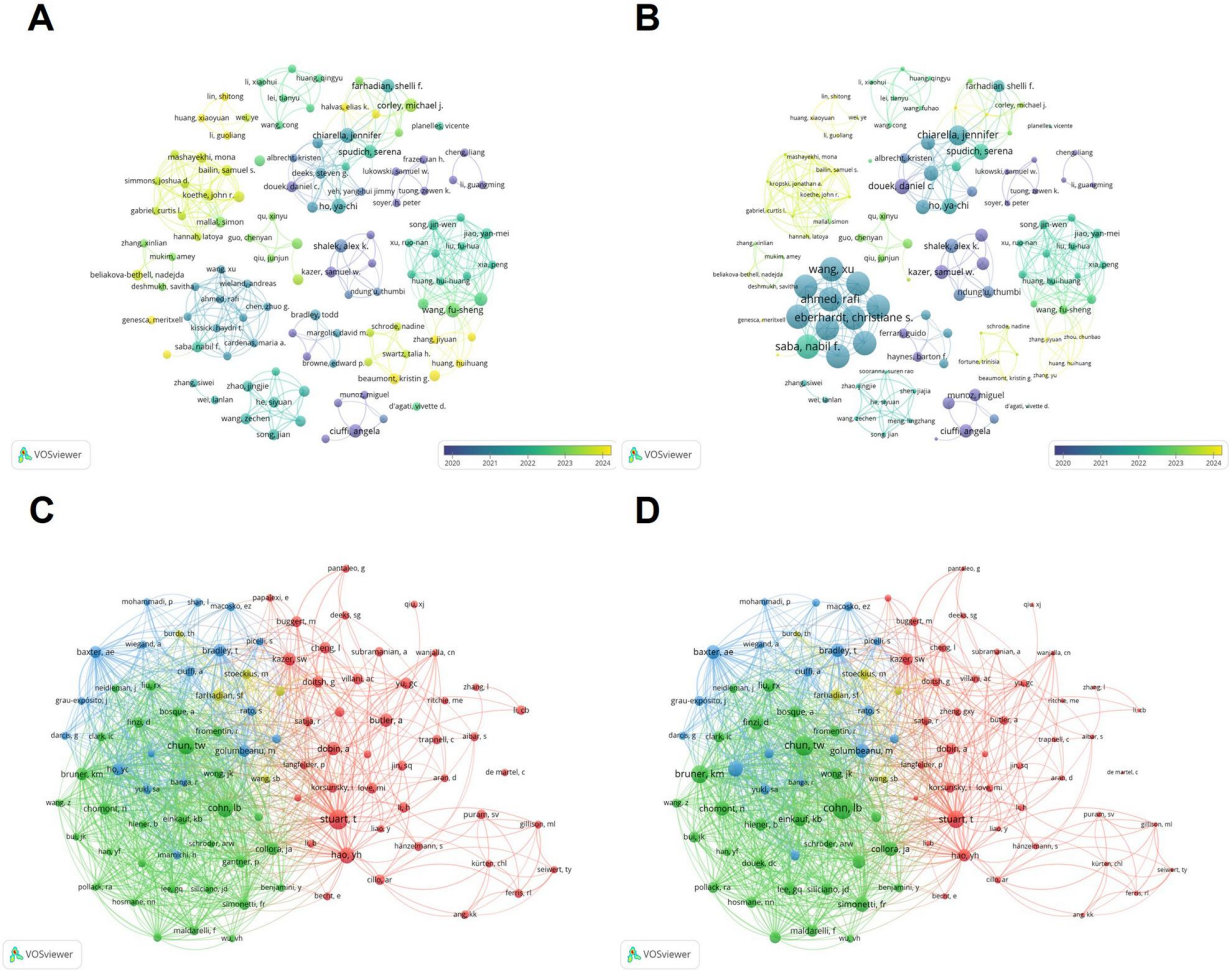
Analysis of authors contributing to research on single-cell sequencing and STIs. Co-occurrence network visualization of authors based on VOSviewer, with nodes sized by documents **(A)** and citation counts **(B)**, respectively. Co-citation network visualization of co-cited authors based on VOSviewer, with nodes sized by co-citation frequency **(C)** and total link strength **(D)**, respectively.

A total of 1,320 authors contributed to the included publications in this study, of whom 116 authors published at least 2 papers. In terms of citation volume (Figure 5B), the research team led by scholars including Saba NF, Wang X, and Ahmed R received the highest citation counts for their works. Regarding publication output (Figure 5A and Table 2), Chiarella J, Ho YC, and Wang FS were the most prolific authors, each publishing five papers. Following closely were six scholars, namely Spudich S, Shalek AK, Ciuffi A, Farhadian SF, Zhang C, and Corley MJ, each of whom published 4 relevant research papers. Notably, Chinese scholars such as Huang XY, Lin ST, Huang HH, and Zhang JY have demonstrated their ability to publish relatively recent research, indicating their capacity for rapid exploration and application in this emerging research areas.

**Table 2:** Top 10 authors and co-cited authors contributed to the study of single-cell sequencing and sexually transmitted infections.

Rank	Author	Count	Citation	Rank	Co-cited author	Co-citation	TLS
1	Chiarella J	5	255	1	Stuart T	41	1,218
2	Ho YC	5	178	2	Chun TW	33	2,033
3	Wang FS	5	75	3	Cohn LB	32	1,808
4	Spudich S	4	175	4	Hao YH	29	725
5	Shalek AK	4	149	5	Dobin A	22	549
6	Ciuffi A	4	136	6	Butler A	21	437
7	Farhadian SF	4	112	7	Kazer SW	20	716
8	Zhang C	4	75	8	Bruner KM	19	1,154
9	Corley MJ	4	32	9	Collora JA	19	861
10	Saba NF	3	362	10	Bradley T	19	814

TLS, total link strength.

“Co-cited authors” refers to authors who are cited together in one or more subsequent publications. Among the 6,562 co-cited authors identified, 96 authors were co-cited at least 8 times. As shown in Figure 5C and Table 2, Stuart T ranked first with 41 co-citations, followed by Chun TW (*n* = 33), Cohn LB (*n* = 32), Hao YH (*n* = 29), and Dobin A (*n* = 22). Total link strength, which measures the degree of association between nodes, was used to assess the academic influence of scholars. As illustrated in Figure 5D, Cohn LB, Chun TW, Bruner KM, and Stuart T exhibited the highest total link strength, suggesting that their works are widely referenced and closely linked within this research community.

### 3.4 Analysis of journals

**Figure 6:**
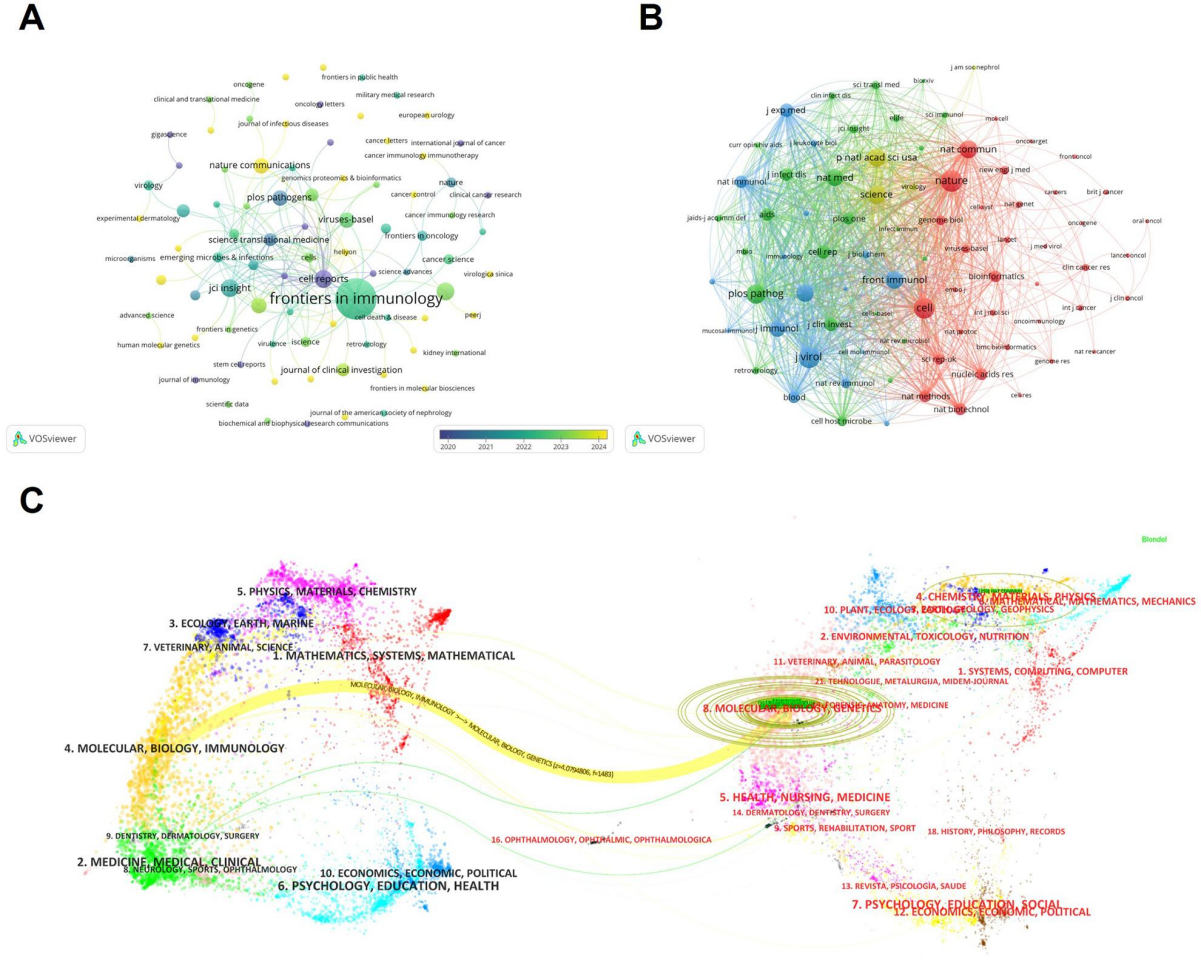
Analysis of journals related to single-cell sequencing and STIs. **(A)** Network visualization of source journals by VOSviewer. Each node represents a journal, and node size is positively correlated with its number of publications. **(B)** Network visualization of co-cited journals by VOSviewer. Each node represents a cited journal, node size is positively correlated with citation frequency, and the link between two nodes indicates that two journals were co-cited in the same article. **(C)** Dual-map overlay of journals from CiteSpace. The left side shows citing journals, while the right side displays cited journals, with curves representing citation relationships between journals.

A total of 85 journals have published papers related to single-cell sequencing and STIs (Figure 6A). Among the top 10 key journals ranked by publication volume (Table 3), 70% are from the United States, with the remaining 20% from Switzerland and 10% from the United Kingdom. FRONTIERS IN IMMUNOLOGY published the most relevant articles (*n* = 20), followed by CELL REPORTS, JCI INSIGHT, and JOURNAL OF MEDICAL VIROLOGY, each with 5 publications. The average citations per item (ACI) is a widely used metric to assess the academic influence of journals in a given field, where higher ACI values reflect stronger scholarly impact. SCIENCE TRANSLATIONAL MEDICINE (*n* = 3, ACI = 55.33) and CELL REPORTS (*n* = 5, ACI = 43) exhibited outstanding academic influence in this research domain. We further analyzed the cited journals in this field. Supplementary Table S5 lists the top 10 journals with the highest co-citation frequencies, and the network of co-cited journals was visualized using VOSviewer (Figure 6B). The top five co-cited journals are NATURE (*n* = 340), JOURNAL OF VIROLOGY (*n* = 329), CELL (*n* = 318), PLOS PATHOGENS (*n* = 282), and SCIENCE (*n* = 256). In addition, we conducted a dual-map overlay analysis of journals using CiteSpace (Figure 6C). The left side shows citing journals, while the right side displays cited journals, with curves representing citation relationships between journals. A distinct citation path (in yellow) was identified, indicating that studies published in Molecular/Biology/Genetics journals are frequently cited by Molecular/Biology/Immunology journals.

**Table 3:** Top 10 journals publishing articles about single-cell sequencing in sexually transmitted infections.

Rank	Journal	NP	NC	ACI	IF (2023)	H-index
1	FRONTIERS IN IMMUNOLOGY	20	193	9.65	5.7	7
2	CELL REPORTS	5	215	43.00	7.5	5
3	JCI INSIGHT	5	121	24.20	6.3	3
4	JOURNAL OF MEDICAL VIROLOGY	5	50	10.00	6.8	4
5	PLOS PATHOGENS	4	75	18.75	5.5	3
6	VIRUSES-BASEL	4	20	5.00	3.8	2
7	CURRENT OPINION IN HIV AND AIDS	4	14	3.50	4.5	2
8	NATURE COMMUNICATIONS	4	6	1.50	14.7	1
9	SCIENCE TRANSLATIONAL MEDICINE	3	166	55.33	15.8	3
10	JOURNAL OF CLINICAL INVESTIGATION	3	25	8.33	13.3	2

NP, number of publications; NC, number of citations; ACI, average citations per item.

### 3.5 Analysis of references and documents

**Figure 7:**
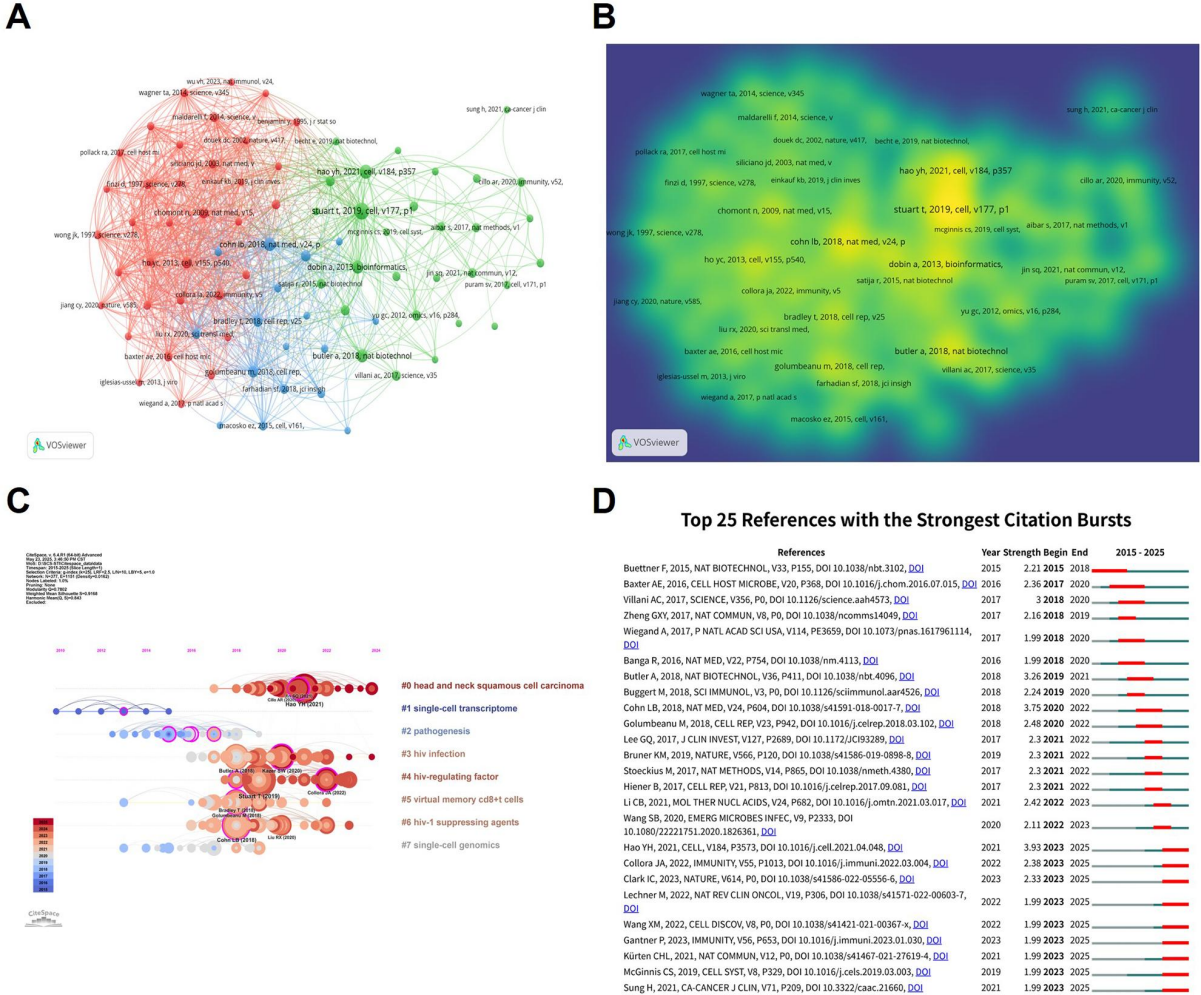
Analysis of references associated with single-cell sequencing and STIs. **(A)** Co-citation network of references based on VOSviewer. Each node represents a reference, with node size positively correlated with the citation frequency, and the link between two nodes represents two references cited in the same article. **(B)** The density visualization of co-cited references based on VOSviewer, with brighter colors indicating higher citation frequencies. **(C)** Timeline view of thematic research topics based on references. Cluster labels on the right of the map summarize core themes of the corresponding clustered literature, revealing major research directions. Purple indicates earlier emergence, while red indicates more recent development. **(D)** Top 25 references with the strongest citation bursts from CiteSpace (*γ* = 0.8). The blue bars indicate the period in which the reference has been published, and the red bars represent bursts of citation frequency.

A total of 7,809 references were co-cited, of which 81 were co-cited over 7 times. The co-citation network of references was constructed using VOSviewer (Figure 7A), while Figure 7B presents their density visualization in a clearer manner, with brighter colors indicating higher citation frequencies. Table 4 details the top 10 most co-cited references. The most frequently co-cited reference is an article by Stuart T et al., published in Cell in 2019, titled “Comprehensive Integration of Single-Cell Data”. This study developed a strategy to “anchor” diverse datasets together, enabling both cross-technology integration and multimodal data integration (25). The second most co-cited reference, “Integrated analysis of multimodal single-cell data” by Hao YH et al., introduced a “weighted-nearest neighbor” analysis method to understand the relative utility of each data type per cell, thereby achieving unified analysis of multimodal data (26). These 10 references focus on single-cell sequencing technologies and delve into their applications in multi-omics data integration and HIV research. Data integration remains a key challenge in single-cell analysis, leading to the development of various multimodal data integration and analysis strategies, such as Seurat (27), the “anchoring” strategy (25), and “weighted-nearest neighbor” analysis (26). In HIV research, latent viral reservoirs represent a major barrier to eradication. Multiple studies have focused on elucidating the characteristics of latently infected cells (13), the transcriptional heterogeneity of latent and reactivated cells (12), the role of host transcriptional regulation (28), mechanisms of viral persistence (29), and the size of latent viral reservoirs (30). These highly co-cited works provide important methodological references for the application of single-cell sequencing in STI-related research.

**Table 4:** Top 10 most co-cited references associated with single-cell sequencing and sexually transmitted infections.

Rank	Title	First author (Year)	Source	Citation	DOI
1	Comprehensive Integration of Single-Cell Data	Stuart T (2019)	CELL	35	10.1016/j.cell.2019.05.031
2	Integrated analysis of multimodal single-cell data	Hao YH (2021)	CELL	24	10.1016/j.cell.2021.04.048
3	Clonal CD4+ T cells in the HIV-1 latent reservoir display a distinct gene profile upon reactivation	Cohn LB (2018)	NAT MED	23	10.1038/s41591-018-0017-7
4	STAR: ultrafast universal RNA-seq aligner	Dobin A (2013)	BIOINFORMATICS	22	10.1093/bioinformatics/bts635
5	Integrating single-cell transcriptomic data across different conditions, technologies, and species	Butler A (2018)	NAT BIOTECHNOL	21	10.1038/nbt.4096
6	Single-Cell RNA-Seq Reveals Transcriptional Heterogeneity in Latent and Reactivated HIV-Infected Cells	Golumbeanu M (2018)	CELL REP	17	10.1016/j.celrep.2018.03.102
7	Replication-competent noninduced proviruses in the latent reservoir increase barrier to HIV-1 cure	Ho YC (2013)	CELL	17	10.1016/j.cell.2013.09.020
8	Single-Cell Analysis of Quiescent HIV Infection Reveals Host Transcriptional Profiles that Regulate Proviral Latency	Bradley T (2018)	CELL REP	16	10.1016/j.celrep.2018.09.020
9	HIV reservoir size and persistence are driven by T cell survival and homeostatic proliferation	Chomont N (2009)	NAT MED	16	10.1038/nm.1972
10	Integrated single-cell analysis of multicellular immune dynamics during hyperacute HIV-1 infection	Kazer SW (2020)	NAT MED	16	10.1038/s41591-020-0799-2

When a set of publications is repeatedly cited, conceptual clusters are formed (31). To gain deeper insights into the development trends and key directions of the research field, Figure 7C presents the timeline view of the top eight clusters identified by CiteSpace. Here, a Modularity *Q* value of 0.7802 and a Mean Silhouette value of 0.9168 indicate significant clustering structure and excellent cluster robustness. Cluster labels on the right side of the map were automatically generated by CiteSpace using the log-likelihood ratio (LLR) algorithm, which extracts representative terms from the keywords of the cited references. These labels serve as algorithmic summaries of the dominant themes within each cluster. With this in mind, head and neck squamous cell carcinoma (HNSCC) (Cluster #0) and HIV-regulating factor (Cluster #4) emerged as clusters with a marked increase in citation activity in recent years, suggesting that these topics have attracted growing research attention. In contrast, single-cell transcriptome (Cluster #1), pathogenesis (Cluster #2), and single-cell genomics (Cluster #7) constitute foundational research themes that emerged earlier in the research timeline.

Analyzing reference bursts can provide critical insights into research hotspots and emerging trends during specific periods (32). Figure 7D shows the top 25 references with the strongest citation bursts. The first co-citation burst occurred in 2015, for the paper titled “Computational analysis of cell-to-cell heterogeneity in single-cell RNA-sequencing data reveals hidden subpopulations of cells” (33). The paper with the strongest burst (strength = 3.93) was published by Hao YH et al. in Cell in 2021 (26), with the burst lasting from 2023 to 2025. Notably, 9 references experienced bursts during 2023–2025, reflecting emerging themes and research frontiers in this field. Of these, 4 are related to HIV infection, and 2 are related to HPV-associated cancers.

Furthermore, Supplementary Table S6 lists the top 10 most cited articles in this field, which have received wide attention from researchers. Among them, 7 are related to HIV infection, and 3 are associated with HPV-related cancers. The top two high-impact papers, both published in 2021 in the premier journal Nature, were the result of a collaboration between the research groups of Rafi Ahmed and Andreas Wieland at Emory University School of Medicine (USA). The research teams focused on the immune response mechanisms in patients with HPV-positive HNSCC, conducting in-depth analyses from two dimensions: HPV-specific CD8^+^ T cells (34) and HPV-specific B cells (35). The former detailed the differentiation trajectories and functional phenotypes of HPV-specific T cells in the tumor microenvironment (TME), while the latter revealed the critical role of B cells in the tumor immune regulatory networks.

### 3.6 Analysis of keywords

**Figure 8:**
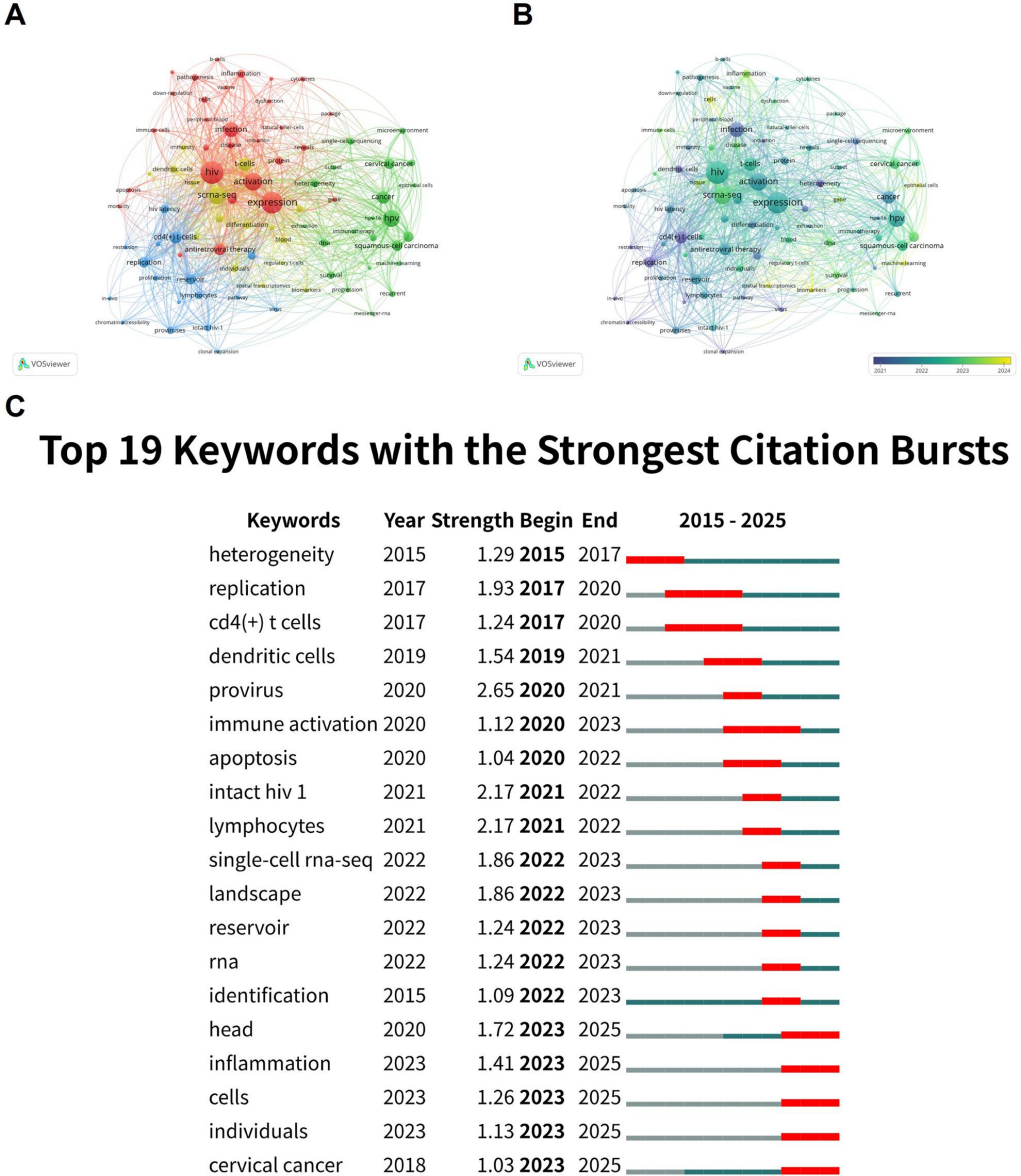
Analysis of keywords related to single-cell sequencing in STIs. **(A)** Co-occurrence network of keywords from VOSviewer. Each node represents a keyword, with node size positively correlated with its occurrence frequency, and the link between two nodes indicates that two keywords appeared together in the same article. Different node colors represent different thematic clusters identified by the algorithm. **(B)** Average year map of keywords from VOSviewer. Each node represents a keyword, with yellow nodes representing newer and frequently occurring keywords, while purple nodes denote keywords from relatively earlier studies. **(C)** Top 19 keywords with the strongest citation bursts based on CiteSpace (*γ* = 0.4). Keywords marked with red bars indicate a sudden increase in usage frequency during that period, while blue bars represent relatively inactive periods.

Keywords in academic papers represent core research themes and priorities. An in-depth analysis of keywords facilitates the identification of emerging research trends and focal points within a discipline. Among 868 extracted keywords, 84 met the threshold of occurring at least three times. As shown in Figure 8A and Table 5, the top 10 most frequent keywords are “hiv”, “expression”, “scrna-seq”, “activation”, “hpv”, “infection”, “t-cells”, “cancer”, “cd4(+) t-cells”, and “antiretroviral therapy”. Node colors also denote distinct thematic clusters, with keywords categorized into 4 clusters. To understand the correlations between keywords, we ranked terms within each cluster by frequency, as shown in Supplementary Table S7. Analyzing keyword clusters allows us to identify prevailing research domains, enabling researchers to gain more detailed insights into each focal area. Next, we analyzed these 4 clusters in sequence.

**Table 5:** Top 20 keywords in terms of occurrences.

Rank	Keyword	Occurrence	TLS
1	HIV	51	569
2	Expression	47	509
3	scRNA-seq	35	390
4	Activation	34	363
5	HPV	32	309
6	Infection	26	290
7	t-Cells	21	228
8	Cancer	19	204
9	Cd4(+) t-cells	18	226
10	Antiretroviral therapy	17	186
11	Replication	15	170
12	Squamous-cell carcinoma	15	143
13	Cervical cancer	15	142
14	Reservoir	14	185
15	Head and neck cancer	14	144
16	Responses	13	120
17	HIV latency	10	136
18	Inflammation	9	106
19	Identification	9	103
20	Protein	9	98

TLS, total link strength.

The red cluster (Cluster #1) encompasses core keywords such as “hiv”, “expression”, “activation”, “infection”, “antiretroviral therapy”, “responses”, “inflammation”, “protein”, “pathogenesis”, “disease”, “mycobacterium-tuberculosis”, “immune cells”, and “apoptosis”, suggesting that the cluster is likely related to research on HIV infection, immune responses, and pathogenic mechanisms. It also likely involves complex issues such as co-infection with *Mycobacterium tuberculosis*.

The green cluster (Cluster #2) includes keywords such as “hpv”, “cancer”, “squamous-cell carcinoma”, “cervical cancer”, “head and neck cancer”, “heterogeneity”, “survival”, “landscape”, “microenvironment”, and “recurrent”, indicating that its research emphasis lies in the heterogeneity of HPV-associated cancers, TME, precision therapy, and prognostic prediction.

The blue cluster (Cluster #3) features keywords such as “cd4(+) t-cells”, “replication”, “reservoir”, “hiv latency”, “identification”, “proviruses”, “lymphocytes”, “intact hiv-1”, “proliferation”, and “persistence”. This points to a primary focus on HIV latent reservoirs and viral persistence.

The yellow cluster (Cluster #4) incorporates keywords such as “scrna-seq”, “t-cells”, “macrophages”, “dendritic cells”, “differentiation”, “individuals”, “receptor”, “memory”, “immunity”, “tissue”, “biomarkers”, “antigen”, and “spatial transcriptomics”. This likely centers on immune profiling via scRNA-seq and spatial transcriptomics.

Figure 8B illustrates the prevalence of keywords over time, with yellow nodes representing newer and frequently occurring keywords, while purple nodes denote keywords from relatively earlier studies. The latest emerging keywords in this field include “spatial transcriptomics”, “epithelial cells”, “biomarkers”, and “machine learning”. Among the top 19 keywords with the most significant citation surges (Figure 8C), we focused on those with important research significance. The top 3 keywords with the strongest burst intensity are “provirus” (2.65), “intact hiv 1” (2.17), and “lymphocytes” (2.17). The three keywords with the longest burst duration are “cd4(+) t cells” (2017–2020), “replication” (2017–2020), and “immune activation” (2020–2023). Recently, “head” (very likely referring to HPV-associated head and neck cancer), “inflammation”, “cells”, “individuals”, and “cervical cancer” have shown increased research attention and may represent emerging topics of interest.

### 3.7 Clinical progress analysis

To evaluate the clinical translation progress of single-cell sequencing in STIs, we systematically queried ClinicalTrials.gov using the preset search strategy. A total of 9 clinical trials that explicitly applied single-cell sequencing technologies in STI-related research were identified, all of which were focused on HIV research (Supplementary Table S8). scRNA-seq was the most widely adopted technology, applied in 7 of the 9 included trials. Additional single-cell approaches included: scATAC-seq, which was paired with scRNA-seq in trial NCT06034314; single-cell combined epigenomic and transcriptomic profiling in trial NCT04920539; and single proviral sequencing in trial NCT02641756. Regarding study design, 5 trials were interventional and 4 were observational. Among the 5 interventional trials, two were classified as Phase 1 (NCT06034314, NCT04920539), one was Phase 1/2 (NCT05668026), one was Phase 4 (NCT05209867), and one trial had no specified phase designation (NCT02641756). The start dates of the included trials spanned from 2016 to 2024. The first relevant trial was initiated in 2016, and only one additional trial was launched in the following 4 years, reflecting very limited early adoption. Since 2022, the number of newly launched trials has increased markedly: 2 trials started in 2022, 3 in 2023, and 2 in 2024. This upward trend indicates that single-cell sequencing is gaining increasing attention in STI clinical research. The sample sizes of included trials were generally small, ranging from 4 to 320 participants, and only 1 study has reported results to date (36). Geographically, all included trials were conducted in high-income settings. Notably, none of the registered clinical trials applied single-cell sequencing to other STI pathogens such as HPV, *Neisseria gonorrhoeae*, or *Chlamydia trachomatis*. Collectively, these findings consistently underscore that the clinical translation of single-cell sequencing in STI research remains at an early exploratory stage, characterized by narrow research scope, insufficient clinical validation, and limited population generalizability.

### 3.8 Cross-database validation analysis

To assess the reliability of the WoSCC-based dataset, a cross-database validation analysis was conducted using the PubMed/MEDLINE database. Key findings are summarized as follows. First, the literature overlap rate reached 69% relative to the WoSCC dataset and 74% relative to the PubMed/MEDLINE dataset, indicating substantial common ground while each database contains unique records (Supplementary Table S2). Second, annual publication trends were highly consistent across the two databases (Spearman's *ρ* = 0.96, *p* < 0.0001; Supplementary Figure S1). Third, country rankings showed identical top four most productive countries (USA, China, Switzerland, Australia), with only minor rank shifts among lower positions (Supplementary Table S3). Fourth, the top 10 most productive journals showed a 90% overlap, with FRONTIERS IN IMMUNOLOGY ranked first in both databases (Supplementary Table S4). Overall, these findings demonstrate that although WoSCC and PubMed/MEDLINE differ in the coverage of certain clinical and regional journals, the core research landscape is consistent across the two databases. Therefore, using WoSCC alone will not introduce significant coverage bias or affect the main conclusions of this study.

## 4 Discussion

### 4.1 General information

To systematically investigate the research landscape and emerging hotspots of single-cell sequencing in STIs, this study integrated several advanced analysis and visualization tools including Microsoft Excel 2021, VOSviewer, CiteSpace, and the Bibliometrix R package. The analysis covered publication volume, contributing countries/regions, institutions, authors, journals, references, and keywords. A total of 148 publications were retrieved from the WoSCC, spanning the period from January 1, 2015 to May 1, 2025. These publications were contributed by 1,320 researchers from 385 institutions across 31 countries/regions and were published in 85 distinct journals.

The bibliometric analysis showed that research at the intersection of single-cell sequencing and STIs has entered an accelerated development phase since 2020, with a steady growth trend in scholarly output. Through quantitative analysis of the academic contributions of countries and institutions, the landscape of core research forces in this field was clarified. The United States and China have emerged as the twin engines driving the field, demonstrating distinct advantages in research productivity. In particular, the United States maintained a central hub position within the global research network by virtue of its extensive international collaborations. CiteSpace analysis identified the top 10 institutions by publication volume, of which 9 were from the United States and 1 was from China. Notably, the National Institutes of Health (NIH)—USA and the Chinese Academy of Sciences ranked high in both publication quantity and centrality metrics, underscoring their pivotal roles.

In addition, the Veterans Health Administration (VHA) and the US Department of Veterans Affairs experienced a significant citation burst during 2023–2025. These two institutions have co-authored 9 publications in this domain, among which the research article by Buggert M et al. received the highest citation count (121 citations). The study revealed that CD8^+^ T cells in lymphoid tissues of HIV-infected individuals exhibit a distinctive molecular profile of tissue-resident memory T cells. Remarkably, the frequency of such HIV-responsive lymphoid tissue-resident CD8^+^ T cells is significantly increased in HIV elite controllers [ECs; defined as individuals who can spontaneously control HIV replication without receiving antiretroviral therapy (ART)] (37). The authors suggested that these cells may play a role in inhibiting HIV replication within lymphoid tissues, and proposed this as a potential mechanism contributing to HIV persistence and as a possible target for immunotherapeutic strategies.

By focusing on the research achievements of outstanding scholars, we can accurately grasp the frontier progress in this field. Stuart T (Genome Institute of Singapore) has the highest number of co-citations, while Jennifer Chiarella (Yale University), Ho Ya-Chi (Yale University), and Wang Fu-Sheng (Chinese People's Liberation Army General Hospital) are the top three most prolific authors. Stuart T et al. published a paper in 2019, introducing an innovative single-cell data integration system. This system achieved a breakthrough in cross-modal integration across single-cell transcriptomic, proteomic, epigenomic, and spatially resolved datasets (25). This publication has received the highest number of co-citations (35 times) among the references in our analysis, reflecting its influence in the field.

Jennifer Chiarella specializes in neuroinfectious diseases, with primary research focused on HIV drug resistance and the development of various methods for detecting low-abundance drug-resistant viral variants. Her team's latest study, which conducted single-cell transcriptome and T-cell receptor sequencing analyses on cerebrospinal fluid and blood samples from HIV-infected individuals receiving ART, revealed that most HIV-1 RNA-positive cells are CD4^+^ central memory T cells. Additionally, the frequency of infected cells in cerebrospinal fluid is higher than that in blood, and 22% of infected T-cell clones are shared between cerebrospinal fluid and blood. These findings suggest that tissue migration and clonal expansion of infected T cells maintain viral reservoirs in the central nervous system (38).

Ho Ya-Chi and his colleagues focus on understanding HIV-1 persistence and HIV-1-induced immune dysfunction using single-genome and single-cell approaches. In 2020, they developed the single-cell HIV-1 SortSeq technology to isolate rare infected cells from HIV-1-infected individuals with viral suppression, and found that aberrant expression of cancer-related genes driven by HIV-1 at integration sites is a mechanism underlying HIV-1 persistence (39). Furthermore, they utilized single-cell multi-omics technology (ECCITEseq) to capture surface protein expression, cellular transcriptome, HIV-1 RNA, and T cell receptor (TCR) sequences in the same single cell, revealing that HIV-1 preferentially resides in GZMB^+^ cytotoxic Th1 effector memory cell clones. Meanwhile, the study identified antigen stimulation, TNF responses, and cytotoxic T cell responses as key drivers of clonal expansion of HIV-1-infected cells (40). This study characterized the single-cell transcriptional landscape of HIV-1 RNA^+^ cells in an *in vivo* state without *ex vivo* stimulation for the first time. The study experienced a significant citation burst during 2023–2025, reflecting its broad and sustained influence in the field.

Wang Fu-Sheng primarily focuses on the clinical management and research of critical infectious diseases (viral hepatitis, HIV/AIDS) and refractory severe liver diseases. The article he co-authored as a corresponding author in Cell Discovery, entitled “Global transcriptomic characterization of T cells in individuals with chronic HIV-1 infection”, showed a significant citation burst during 2023–2025 (41). His latest research revealed that the GZMK^+^ GZMB^+^ CD8^+^ T cell subset is significantly enriched in HIV-1-infected individuals through integrating scRNA-seq, TCR-seq, flow cytometry, and functional assays. This subset, characterized by high proliferation, activation, inflammatory responses, and clonal switching, is one of the endpoints of CD8^+^ αβ T cell differentiation. Its quantity is negatively correlated with the CD4/CD8 ratio, and positively correlated with HIV DNA and inflammatory factor levels, suggesting its role in HIV-associated systemic inflammation (42). Moreover, his research team also confirmed that highly clonal expansion and the senescent state of CD4^+^ GNLY^+^ T cells may be important factors leading to poor immune reconstitution in HIV-1-infected patients (43). Interestingly, CiteSpace's local author analysis revealed that no author had a centrality score exceeding 0.10, indicating limited scholarly connectivity among researchers. Therefore, we strongly recommend that scholars from the United States, China, and other countries strengthen academic cooperation in the future, break down academic barriers, and promote the development of single-cell sequencing in STIs.

Analysis of publication sources identified FRONTIERS IN IMMUNOLOGY as the most productive journal related to single-cell sequencing and STIs, with a total of 20 articles. Among the top 10 journals, 3 achieved an impact factor exceeding 10 in 2023, namely NATURE COMMUNICATIONS (14.7), SCIENCE TRANSLATIONAL MEDICINE (15.8), and JOURNAL OF CLINICAL INVESTIGATION (13.3). In the co-citation analysis, the journals with the highest co-citation frequencies were NATURE (*n* = 340), JOURNAL OF VIROLOGY (*n* = 329), and CELL (*n* = 318). Analyses of journal sources and citation distribution help identify core journals in the field, which provides important guidance for scholars in selecting appropriate journals for submission in the future.

### 4.2 Hotspots and frontiers

Based on keyword co-occurrence, burst detection and reference co-citation analysis, we identified core research hotspots and emerging frontiers in this field, which are highly concentrated in HIV infection and HPV-associated cancers. Below we interpret the development status and clinical value of these core directions combined with representative studies, to further illustrate the practical significance of our bibliometric findings.

#### 4.2.1 HIV infection: immune mechanism and latent reservoir research

Our keyword clustering analysis identified two core HIV-related research directions: immune response and pathogenic mechanisms, as well as latent reservoirs and viral persistence. Notably, “provirus” showed the highest burst strength among all keywords, indicating that HIV latent reservoir research is the most concerned frontier direction in recent years.

In terms of immune mechanism research, single-cell sequencing has revealed the functional heterogeneity of innate immune cells and adaptive immune cells during HIV infection, which deepens the understanding of viral immune escape and disease progression (44–47). For example, Brouiller et al. demonstrated that among human blood DC subsets, Axl⁺ DCs mount a more robust and broad-spectrum innate immune response upon HIV-1 exposure. This study revealed a dual sensing mechanism of Axl⁺ DCs for HIV-1: one is an NF-κB-mediated program that can induce DCs maturation and effectively activate CD4⁺ T cells; the other is a STAT1/2-mediated program that can activate the type I interferon (IFN) and interferon-stimulated gene (ISG) responses. Importantly, this subset can initiate sensing of HIV-1 even before viral replication, and the entry route of HIV-1 may determine distinct innate sensing pathways in DCs (44). Expanding further to the mucosal barrier, Parthasarathy et al. characterized the genital tract DC population at the single-cell level and explored the immediate responses of DC subsets upon exposure to HIV. The study found that the genital tract CD11c^+^ HLA-DR^+^ myeloid cell population comprises three DC subsets (CD1c^+^ DC2s, CD14^+^ monocyte-derived DCs and CD14^+^ CD1c^+^ DC3s) and two monocyte/macrophage populations, which exhibit distinct functions and phenotypes under steady-state conditions. After HIV exposure, DCs rapidly secrete cytokines and activate non-canonical inflammatory pathways and host restriction factors. Among them, CD14⁺ DCs dominate the secretory antimicrobial response, while CD1c⁺ DC2s activate the inflammasome pathway and IFN response. Identifying subset-specific immediate responses to HIV exposure may facilitate the development of targeted strategies against HIV infection (45). Related studies have also explored the cellular characteristics of incomplete immune reconstitution after antiretroviral therapy, providing potential targets for improving clinical prognosis. For instance, Li et al. reported a reduction in mucosal-associated invariant T (MAIT) cells in immune non-responders [INRs; defined as HIV-infected individuals with incomplete immune reconstitution despite long-term effective antiretroviral therapy (48)], accompanied by mitochondrial dysfunction (49). Jia et al. used single-cell immune repertoire sequencing (scVDJ-seq) and found that B cell receptor (BCR) repertoire diversity is impaired in INRs, with abnormal differentiation of naive B cells (50).

As the core challenge of HIV functional cure, latent reservoir research has maintained high academic attention, which is consistent with the continuous citation burst of related keywords. Two recent pioneering studies have made breakthroughs in identifying rare latent cells using single-cell multi-omics technologies and lineage tracing models, providing critical theoretical support for the “Shock and Kill” strategy. Ho Ya-Chi's team utilized single-cell DOGMA-seq technology, which integrates ATAC-seq, RNA-seq, and surface protein profiling, to simultaneously profile transcription factor accessibility, transcriptomes, surface proteins, HIV-1 DNA, and HIV-1 RNA in memory CD4^+^ T cells from 6 HIV-1-infected individuals during viremia and after suppressive ART. This approach enabled comprehensive characterization of epigenetic, transcriptional, and proteomic landscapes of both latent and transcriptionally active HIV-1-infected cells. The study identified four distinct cellular states in HIV-1-infected cells: cytotoxic CD4^+^ T cells, activated cells, migratory cells, and dying cells. Moreover, the transcription factor IKZF3 was significantly upregulated in two infected cell types and closely associated with cellular proliferation, suggesting that IKZF3 may promote HIV-1 persistence (51). Satija et al. developed the HIV-1-induced lineage tracing (HILT) system, an irreversible labeling model for infected cells in humanized mouse models, which allows detection of rare latently infected cells. Through scRNA-seq of HILT-labeled cells during acute infection and post-ART, they identified distinct transcriptional lineages of CD4⁺ T cells enriched in either active or latent infection states. Furthermore, researchers identified common pathways regulated in both states, including EIF2, Sirtuin, and protein ubiquitination pathways, with key regulators of these pathways (such as *JUN*, *BCL2*, and *MDM2*) showing opposite expression trends between the two states. These differential gene expression programs elucidate mechanisms of HIV persistence across T-cell lineages and infection states, revealing potential drug targets for modulating latency or infected cell survival (52). These studies have pioneered a new paradigm for HIV cure research. The combination of lineage tracing and single-cell multi-omics in future studies is expected to enable precise identification and targeted elimination of latent HIV reservoirs.

#### 4.2.2 HPV-associated cancers: TME heterogeneity and precision therapy

Reference co-citation clustering showed that HPV-associated head and neck squamous cell carcinoma (Cluster #0) has maintained significantly increased citation activity in recent years. Combined with keyword clustering analysis and burst keywords such as “cervical cancer”, it confirms that TME heterogeneity and precision therapy of HPV-associated cancers are another core research hotspot in this field.

Single-cell sequencing technologies deepen our understanding of TME by dissecting its cellular complexity and functional heterogeneity, which is crucial for identifying novel biomarkers and therapeutic targets. By integrating scRNA-seq with spatial transcriptomics, researchers have revealed marked differences in microenvironmental features between HPV^+^ HNSCC and HPV^−^ HNSCC. Lymphoid cells are highly enriched in HPV^+^ HNSCC tumor tissues, and there exist tertiary lymphoid structures (TLS)—such structures are closely associated with good prognosis of patients, suggesting a stronger potential for immune response. In contrast, HPV^−^ HNSCC tumor cells are mostly co-localized with stromal cells such as cancer-associated fibroblasts (CAFs) and endothelial cells, accompanied by reduced immune cell infiltration, which contributes to the formation of an immunosuppressive TME (53). Similarly, through integrating scRNA-seq and spatial transcriptomics, Guo et al. pioneered the characterization of the temporal dynamics and spatial distribution of cell subsets during disease progression from normal cervical tissue to precancerous lesions and finally cervical cancer. The study identified three types of “HPV-associated epithelial cell clusters” unique to normal cervix, high-grade squamous intraepithelial lesions (HSIL) and cervical cancer tissues, respectively. Additionally, hub genes regulating disease progression were identified, including CALML5 and CXCL10, which drive the malignant transformation from HSIL to cervical cancer. Furthermore, the study captured the dynamic process of multiple immune cells from positive response to functional disorder and exhaustion, demonstrating that the cervical microenvironment undergoes dynamic changes of “homeostasis—imbalance—malignancy” during disease progression. These findings provide new targets and insights for accurate diagnosis, precise treatment and prognostic evaluation of cervical cancer (54). Moreover, Yan et al. discovered that compared to HPV^−^ oropharyngeal squamous cell carcinoma (OPSCC), HPV^+^ OPSCC tumor cells display stronger IFN responses and higher expression of major histocompatibility complex class II (MHC-II) molecules. They also identified a CXCL13⁺ CD4⁺ T-cell subset with dual features of follicular helper T cells and pro-inflammatory helper T cells. This T-cell subset establishes extensive crosstalk with HPV^+^ tumor cells, promoting the release of CXCL13 and interferon-gamma (IFN-γ), thereby shaping a pro-inflammatory TME. Notably, high MHC-II expression in HPV^+^ tumor cells and enrichment of CXCL13⁺ CD4⁺ T cells are associated with improved overall survival in OPSCC patients (55).

Single-cell sequencing technologies enable dynamic monitoring of TME remodeling before and after treatment, tracking the functional states of immune cells, and evaluating therapeutic efficacy. Kim et al. reported a single-arm phase 2 trial investigating the neoadjuvant therapeutic effects of pembrolizumab (an immune checkpoint inhibitor), GX-188E (a therapeutic HPV DNA vaccine), and GX-I7 (a long-acting interleukin-7) in patients with resectable HPV-16 and/or 18-positive HNSCC. Integrating single-cell transcriptomics with artificial intelligence-driven spatial profiling, the study found a significant increase in the proportion of T follicular helper cell clusters among tumor-infiltrating immune cells after treatment. Concurrently, CD8⁺ T cell clusters underwent remodeling towards a less exhausted phenotype, characterized by upregulated expression of TCF7 and CD28. Furthermore, the triple therapy significantly increased the density of TILs, completely converting immune-desert and immune-excluded tumors into an inflamed immune phenotype (56). Additionally, utilizing TCR-enriched scRNA-seq, Poppe et al. demonstrated that the triple therapy (HPV vaccine PDS0101, tumor-targeting immune cytokine NHS-IL12, and class I histone deacetylase inhibitor Entinostat) markedly enhanced the infiltration of total and HPV-specific CD8^+^ T cells in tumors, boosted their cytotoxicity, and induced the expansion of unique CD8⁺ T cell clones, with the cytotoxic transcriptional profiles of these clones being significantly enriched. Meanwhile, this therapy can promote the differentiation of tumor-associated macrophages (TAMs) towards pro-inflammatory and anti-tumor M1-like phenotypes, and activate inflammatory and immune-related signaling pathways. These effects collectively shaped a highly immunologically active and pro-inflammatory TME (57).

### 4.3 Challenges and future directions

Despite the rapid advances in single-cell research on STIs, substantial research imbalances persist in this field. Most studies (over 90%) focus on HIV and HPV-related cancers, while research on other common STI pathogens, such as *Neisseria gonorrhoeae*, *Chlamydia trachomatis*, and HSV-2, remains limited. This discrepancy is driven by several factors. From the perspective of scientific demand, HIV, as a global pandemic and incurable chronic infectious disease, presents high complexity in its core scientific issues, such as the mechanisms of latent reservoir establishment, immune evasion pathways, and treatment resistance. Meanwhile, HPV infection, as a well-established carcinogenic factor, involves complex host—virus—microenvironment interactions during the carcinogenesis process of cervical cancer, anal cancer, and other cancers (4, 5). With their unique advantages in dissecting cellular heterogeneity and tracking the evolution of immune microenvironments, single-cell sequencing technologies has naturally become critical tools to address these core issues. From the perspective of resource foundations, long-term research on HIV and HPV has accumulated abundant knowledge and resources, including a large number of clinical samples, cell lines, and animal models, which provide a solid basis for related single-cell studies. In contrast, research on other STI pathogens has largely relied on traditional microbiological and molecular biology techniques. Moreover, the clinical demand for single-cell sequencing is considered lower for these pathogens, as most associated infections can be effectively treated with antibiotics, despite the escalating threat of antimicrobial resistance. Notably, the “Matthew effect” in resource allocation has further exacerbated the imbalance. Research funding, scientific talents, and technical platforms are increasingly concentrated in the high-profile fields of HIV and HPV research, forming a self-reinforcing research cycle. Consequently, studies on other STI pathogens face persistent bottlenecks, resulting in a continuously widening research disparity.

Despite existing challenges, single-cell sequencing technologies have exhibited potential in investigating other STI pathogens beyond HIV and HPV. In 2019, Hayward RJ et al. applied scRNA-seq technology to chlamydia infection research for the first time, revealing significant transcriptional heterogeneity in host epithelial cells during the early stage of *Chlamydia trachomatis* infection and identifying the temporal expression characteristics of genes related to metallothioneins, cell cycle regulation, and innate immunity (58). Subsequently, Mercado et al. integrated scRNA-seq with TCR repertoire profiling, and identified the transcription factor BHLHE40 as a key molecular driver for the differentiation of protective multifunctional CD4⁺ T cells against chlamydia in the female reproductive tract (59). Brothwell et al. integrated scRNA-seq and spatial transcriptomics to construct the first high-resolution single-cell immune atlas of *Haemophilus ducreyi* infection, revealing that the spatial co-localization network of IFN-I-activated macrophages and T/NK cell-antigen-presenting cells in the pustule microenvironment constitutes a key immune feature (60). Additionally, Chen et al. utilized scRNA-seq to profile a high-resolution single-cell atlas of peripheral blood immune cells in patients with genital herpes during recurrence, systematically uncovering the characteristics of immune cell activation, abnormal signaling pathways, and intercellular interaction mechanisms during recurrence. These findings provide a novel perspective for understanding the systemic immune response to local HSV-2 reactivation and offer crucial theoretical support for targeted immunotherapy strategies (such as regulating the TLR/IL-17 pathway) (61). These research achievements highlight the irreplaceable role of single-cell sequencing in dissecting the spatiotemporal dynamics of immune responses to STIs. With the decreasing cost of technologies and the popularization of automated platforms, single-cell sequencing is expected to unleash their full potential in research on a broader spectrum of STI pathogens.

While this bibliometric analysis reveals research trends, the primary literature has notable limitations. Most included studies have small sample sizes, lack of validation cohorts, descriptive nature, and minimal clinical translation, which should be considered when interpreting our results. Future studies should prioritize larger, multicenter cohorts, standardized protocols, and integration with functional and clinical endpoints. Beyond these general recommendations, systematic multi-dimensional strategies are needed in the future. First, it is essential to deepen the integration of multi-modal technologies. scRNA-seq provides only static transcriptomic information and cannot capture spatial information or multi-omics data, and suffers from batch effects and high costs (11). The combination of scRNA-seq with other omics analyses is a highly active research field at present. By integrating information from transcriptomics, epigenomics, proteomics, and other omics, researchers can gain a more comprehensive understanding of the functions and interaction modes of individual cells in complex biological systems. In the spatial dimension, integrating single-cell multi-omics with spatial transcriptomic information enables the dissection of “cell-cell” and “cell-matrix” interaction networks while preserving the spatial location information of cells, thereby fully understanding the dynamic changes of cells in specific spatial positions. In the temporal dimension, in addition to longitudinal studies, combining time-resolved technologies can construct more accurate models of cellular processes, which is expected to track the dynamic changes of cells during pathogen invasion, drug intervention, and other processes. For instance, Live-seq uses fluidic force microscopy (FluidFM) to extract cytoplasmic RNA from living cells, allowing longitudinal monitoring of transcriptomic and phenotypic dynamics in individual macrophages before and after lipopolysaccharide (LPS) stimulation (62). Furthermore, integrating organoid technology with single-cell sequencing can simulate tissue microenvironment of STIs *in vitro*, offering powerful support for drug screening and pathological mechanism studies. Second, AI-driven translation should be promoted. AI and machine learning can be used to efficiently process the massive datasets generated by single-cell sequencing, identify complex patterns that are difficult to detect with traditional methods, and accurately predict therapeutic responses and biomarkers. Third, there is an urgent need to improve technical accessibility. In the future, more economical and higher-throughput single-cell capture technologies should be developed, and data analysis workflows should be optimized to enhance the accessibility and reliability of single-cell sequencing in STIs. Through breakthroughs in the above directions, single-cell sequencing in STIs will move from single-dimensional molecular analysis to multi-dimensional and dynamic systems biology research, providing new scientific foundations for infection prevention and control, vaccine development, and precision therapy.

### 4.4 Limitations

Although this study comprehensively identified the research hotspots and emerging trends of single-cell sequencing in STIs through bibliometric and visual analyses, several limitations should be acknowledged. First, this study only included English-language publications and did not cover research written in other languages such as Chinese. This may lead to the neglect of some region-specific research and introduce a degree of language bias. Second, the data source was limited to the WoSCC database, which might exclude relevant articles available in other important databases and result in the omission of certain related studies, particularly for publications from low- and middle-income countries. This coverage bias may underestimate the research output of low- and middle-income countries and miss their region-specific research hotspots. Third, grey literature was not included in this study, which may also cause the omission of relevant research. Despite these limitations, as the most widely used and authoritative database for bibliometric analyses, the WoSCC database contains sufficient high-quality literature to relatively comprehensively reflect the current research landscape in this field.

This study also faces the limitation of a small sample size. Only 148 publications were retrieved over the period from 2015 to 2025, which likely reflects the relatively niche intersection of single-cell sequencing and STIs. The modest sample size may lead to several limitations. First, the stability of cluster structures may be weaker than in large-scale studies, and the addition or removal of a few publications could affect cluster assignments. Second, burst detection is sensitive to extreme values, and incomplete 2025 data may affect the identification of recent burst terms. Third, in collaboration network analysis, connections involving low-frequency countries or institutions may be less reliable. Therefore, our findings should be considered preliminary and exploratory. As the field advances, future validation studies with larger sample sizes will be necessary.

## 5 Conclusion

By systematically analyzing the literature data on single-cell sequencing in STIs, this study comprehensively evaluated publication characteristics across years, countries/regions, research institutions, author groups, and journals, and deeply explored the thematic evolution trends and emerging research hotspots. Our findings highlight the pivotal role of single-cell sequencing (especially scRNA-seq) in advancing STI research. Since 2020, attention to single-cell sequencing in STI research has increased significantly, with a rapid growth in related research outputs. The United States holds a leadership position in this domain, followed closely by China. However, international collaboration remains limited and should be strengthened to drive further progress. Notably, current research focuses primarily on using scRNA-seq to investigate HIV immunopathogenesis and HPV-associated cancers. Moving forward, integrating multi-omics, spatial transcriptomics, and organoid models will likely offer new insights into the complex pathological mechanisms of STIs and pave the way for innovative diagnostic and therapeutic strategies.

## Data Availability

The original contributions presented in the study are included in the article/Supplementary Material, further inquiries can be directed to the corresponding authors.
