# Supplementary material for: Global research landscape and trends of single-cell sequencing in sexually transmitted infections: a comprehensive bibliometric analysis from 2015 to 2025
Source: Front Reprod Health. 2026 Jul 13;8:1817416. doi: 10.3389/frph.2026.1817416 (PMC13402426; doi:10.3389/frph.2026.1817416)
Supplement: Supplementary file 1 [file Table1.docx]

**Global research landscape and trends of single-cell sequencing in sexually transmitted infections: A comprehensive bibliometric analysis from 2015 to 2025**

Cuixiu Wu^1^, Yuanshuo Guo^1^, Na Cui^1^, Yidan Gao^1^, Lijuan Jiang^1^, Haoneng Tang^1^, Qingchun Liang^2, 3*^, Lingli Tang^1*^

^1^ Department of Laboratory Medicine, The Second Xiangya Hospital, Central South University, Changsha, Hunan, China

^2^ Department of Pathology, The Second Xiangya Hospital, Central South University, Changsha, Hunan, China

^3^ Hunan Clinical Medical Research Center for Cancer Pathogenic Genes Testing and Diagnosis, Changsha, Hunan, China

*** Corresponding authors:**

Qingchun Liang; e-mail: 503079@csu.edu.cn

Lingli Tang; e-mail: [linglitang@csu.edu.cn](mailto:linglitang@csu.edu.cn)

**Table S1 Comparison between original search strategy and alternative broad search strategy in WoSCC for sensitivity analysis.**

| Dataset | Set 1 (Final adopted strategy in manuscript) | Set 2 (Alternative broad strategy for sensitivity test only) |
| --- | --- | --- |
| Search query | TS = (“single-cell sequencing” OR “single-cell genom*” OR “single-cell DNA seq*” OR “scDNA-seq” OR “single-cell transcriptom*” OR “single-cell RNA seq*” OR “scRNA-seq” OR “single-cell metabolom*” OR “single-cell proteom*” OR “single-cell epigenom*” OR “scATAC-seq” OR “single-cell omics” OR “single-cell multiomics” OR “single-cell immune profiling”) AND TI = (“sexually transmitted infection*” OR “sexually transmitted disease*” OR “lymphogranuloma venereum” OR “chlamydia trachomatis” OR “genital chlamydia” OR “syphilis” OR “treponema pallidum” OR “HSV-2” OR “genital herpes” OR “genital ulcer” OR “human papillomavirus” OR “HPV” OR “human immunodeficiency virus” OR “HIV” OR “chancroid” OR “haemophilus ducreyi”) | TS = (“single-cell sequencing” OR “single-cell genom*” OR “single-cell DNA seq*” OR “scDNA-seq” OR “single-cell transcriptom*” OR “single-cell RNA seq*” OR “scRNA-seq” OR “single-cell metabolom*” OR “single-cell proteom*” OR “single-cell epigenom*” OR “scATAC-seq” OR “single-cell omics” OR “single-cell multiomics” OR “single-cell immune profiling”) AND TS = (“sexually transmitted infection*” OR “sexually transmitted disease*” OR “lymphogranuloma venereum” OR “chlamydia trachomatis” OR “genital chlamydia” OR “syphilis” OR “treponema pallidum” OR “HSV-2” OR “genital herpes” OR “genital ulcer” OR “human papillomavirus” OR “HPV” OR “human immunodeficiency virus” OR “HIV” OR “chancroid” OR “haemophilus ducreyi”) |
| Time span | 2015/01/01-2025/05/01 | 2015/01/01-2025/05/01 |
| Document types | Article OR Review | Article OR Review |
| Language | English | English |
| Original records | 148 | 294 |
| Valid records after manual screening | 148 | 191 |

**Table S2** Search strategies and basic retrieval results: WoSCC vs. PubMed/MEDLINE.

| Database | WoSCC | PubMed/MEDLINE |
| --- | --- | --- |
| Search query | TS=(“single-cell sequencing” OR “single-cell genom*” OR “single-cell DNA seq*” OR “scDNA-seq” OR “single-cell transcriptom*” OR “single-cell RNA seq*” OR “scRNA-seq” OR “single-cell metabolom*” OR “single-cell proteom*” OR “single-cell epigenom*” OR “scATAC-seq” OR “single-cell omics” OR “single-cell multiomics” OR “single-cell immune profiling”) AND TI=(“sexually transmitted infection*” OR “sexually transmitted disease*” OR “lymphogranuloma venereum” OR “chlamydia trachomatis” OR “genital chlamydia” OR “syphilis” OR “treponema pallidum” OR “HSV-2” OR “genital herpes” OR “genital ulcer” OR “human papillomavirus” OR “HPV” OR “human immunodeficiency virus” OR “HIV” OR “chancroid” OR “haemophilus ducreyi”) | ("single-cell sequencing"[Title/Abstract] OR "single-cell genom*"[Title/Abstract] OR "single-cell DNA seq*"[Title/Abstract] OR "scDNA-seq"[Title/Abstract] OR "single-cell transcriptom*"[Title/Abstract] OR "single-cell RNA seq*"[Title/Abstract] OR "scRNA-seq"[Title/Abstract] OR "single-cell metabolom*"[Title/Abstract] OR "single-cell proteom*"[Title/Abstract] OR "single-cell epigenom*"[Title/Abstract] OR "scATAC-seq"[Title/Abstract] OR "single-cell omics"[Title/Abstract] OR "single-cell multiomics"[Title/Abstract] OR "single-cell immune profiling"[Title/Abstract]) AND ("sexually transmitted infection*"[Title] OR "sexually transmitted disease*"[Title] OR "lymphogranuloma venereum"[Title] OR "chlamydia trachomatis"[Title] OR "genital chlamydia"[Title] OR "syphilis"[Title] OR "treponema pallidum"[Title] OR "HSV-2"[Title] OR "genital herpes"[Title] OR "genital ulcer"[Title] OR "human papillomavirus"[Title] OR "HPV"[Title] OR "human immunodeficiency virus"[Title] OR "HIV"[Title] OR "chancroid"[Title] OR "haemophilus ducreyi"[Title]) |
| Time span | 2015/01/01-2025/05/01 | 2015/01/01-2025/05/01 |
| Document types | Article OR Review | Journal Article OR Review |
| Language | English | English |
| Original records | 148 | 137 |
| Records after deduplication | 148 | 137 |
| Overlap between two databases (Overlap rate) | 102 (68.9%) | 102 (74.5%) |


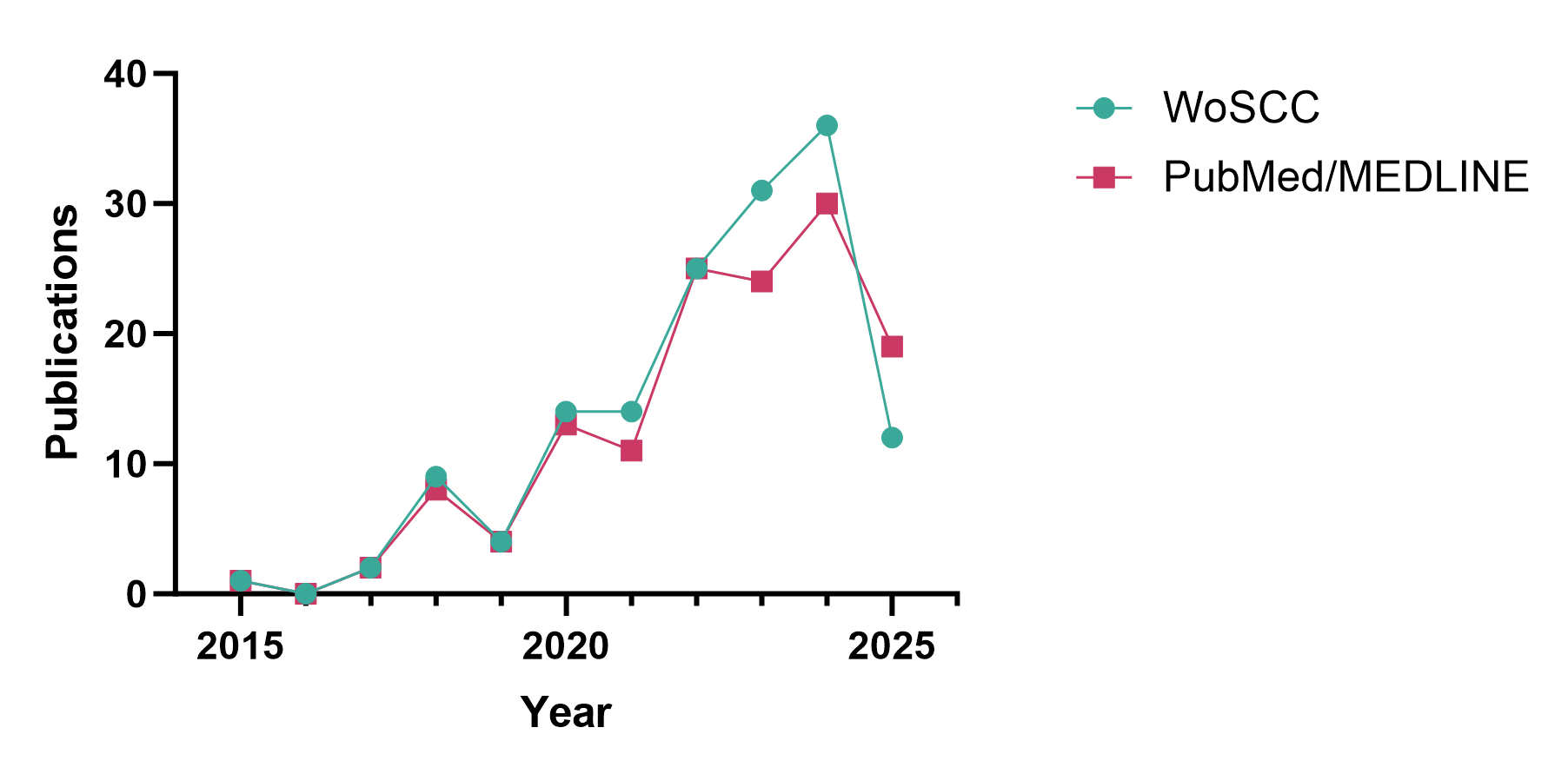
**Figure S1** Annual publication trends from 2015 to 2025: WoSCC vs. PubMed/MEDLINE. The data for 2025 is incomplete. Spearman’s rank correlation coefficient ρ = 0.9613, p < 0.0001 (two-tailed).

**Table S3** Top 10 countries by publication volume: WoSCC vs. PubMed/MEDLINE

| Rank | WoSCC | Count | PubMed/MEDLINE | Count |
| --- | --- | --- | --- | --- |
| 1 | USA | 67 | USA | 51 |
| 2 | CHINA | 46 | CHINA | 40 |
| 3 | SWITZERLAND | 5 | SWITZERLAND | 5 |
| 4 | AUSTRALIA | 4 | AUSTRALIA | 4 |
| 5 | GERMANY | 4 | FRANCE | 3 |
| 6 | FRANCE | 3 | GERMANY | 3 |
| 7 | JAPAN | 3 | JAPAN | 3 |
| 8 | CANADA | 2 | SPAIN | 3 |
| 9 | ITALY | 2 | CANADA | 1 |
| 10 | SPAIN | 2 | ITALY | 1 |

**Table S4** Top 10 source journals by publication volume: WoSCC vs. PubMed/MEDLINE

| Rank | WoSCC | Count | PubMed/MEDLINE | Count |
| --- | --- | --- | --- | --- |
| 1 | FRONTIERS IN IMMUNOLOGY | 20 | FRONTIERS IN IMMUNOLOGY | 24 |
| 2 | CELL REPORTS | 5 | CELL REPORTS | 6 |
| 3 | JCI INSIGHT | 5 | JCI INSIGHT | 5 |
| 4 | JOURNAL OF MEDICAL VIROLOGY | 5 | JOURNAL OF MEDICAL VIROLOGY | 5 |
| 5 | PLOS PATHOGENS | 4 | NATURE COMMUNICATIONS | 4 |
| 6 | VIRUSES-BASEL | 4 | PLOS PATHOGENS | 4 |
| 7 | CURRENT OPINION IN HIV AND AIDS | 4 | VIRUSES | 4 |
| 8 | NATURE COMMUNICATIONS | 4 | CURRENT OPINION IN HIV AND AIDS | 3 |
| 9 | SCIENCE TRANSLATIONAL MEDICINE | 3 | SCIENCE TRANSLATIONAL MEDICINE | 3 |
| 10 | JOURNAL OF CLINICAL INVESTIGATION | 3 | JOURNAL OF CLINICAL INVESTIGATION | 3 |

**Table S5** Top 10 most co-cited journals related to single-cell sequencing and STIs.

| Rank | Co-cited journal | Citations | IF (2023) |
| --- | --- | --- | --- |
| 1 | NATURE | 340 | 50.5 |
| 2 | J VIROL | 329 | 4 |
| 3 | CELL | 318 | 45.6 |
| 4 | PLOS PATHOG | 282 | 5.5 |
| 5 | SCIENCE | 256 | 44.8 |
| 6 | P NATL ACAD SCI USA | 255 | 9.4 |
| 7 | FRONT IMMUNOL | 251 | 5.7 |
| 8 | NAT COMMUN | 250 | 14.7 |
| 9 | IMMUNITY | 240 | 25.5 |
| 10 | NAT MED | 226 | 58.7 |

**Table S6** Top 10 most cited documents about single-cell sequencing in STIs.

| Rank | Title | First author  (Year) | Source | Citations | DOI |
| --- | --- | --- | --- | --- | --- |
| 1 | Functional HPV-specific PD-1^+^ stem-like CD8 T cells in head and neck cancer | Eberhardt CS  (2021) | NATURE | 209 | 10.1038/s41586-021-03862-z |
| 2 | Defining HPV-specific B cell responses in patients with head and neck cancer | Wieland A  (2021) | NATURE | 147 | 10.1038/s41586-020-2931-3 |
| 3 | B Cells Improve Overall Survival in HPV-Associated Squamous Cell Carcinomas and Are Activated by Radiation and PD-1 Blockade | Kim SS  (2020) | CLIN CANCER RES | 146 | 10.1158/1078-0432.CCR-19-3211 |
| 4 | Identification and characterization of HIV-specific resident memory CD8^+^ T cells in human lymphoid tissue | Buggert M  (2018) | SCI IMMUNOL | 121 | 10.1126/sciimmunol.aar4526 |
| 5 | Integrated single-cell analysis of multicellular immune dynamics during hyperacute HIV-1 infection | Kazer SW  (2020) | NAT MED | 95 | 10.1038/s41591-020-0799-2 |
| 6 | Shared transcriptional profiles of atypical B cells suggest common drivers of expansion and function in malaria, HIV, and autoimmunity | Holla P  (2021) | SCI ADV | 85 | 10.1126/sciadv.abg8384 |
| 7 | Elite control of HIV is associated with distinct functional and transcriptional signatures in lymphoid tissue CD8^+^ T cells | Nguyen S  (2019) | SCI TRANSL MED | 85 | 10.1126/scitranslmed.aax4077 |
| 8 | Single-cell RNA sequencing reveals microglia-like cells in cerebrospinal fluid during virologically suppressed HIV | Farhadian SF  (2018) | JCI INSIGHT | 80 | 10.1172/jci.insight.121718 |
| 9 | Human Dendritic Cell Subsets, Ontogeny, and Impact on HIV Infection | Rhodes JW  (2019) | FRONT IMMUNOL | 79 | 10.3389/fimmu.2019.01088 |
| 10 | Single-Cell RNA-Seq Reveals Transcriptional Heterogeneity in Latent and Reactivated HIV-Infected Cells | Golumbeanu M  (2018) | CELL REP | 77 | 10.1016/j.celrep.2018.03.102 |

**Table S7** Cluster of keywords.

| Cluster | Color | Keywords |
| --- | --- | --- |
| 1 | Red | hiv, expression, activation, infection, antiretroviral therapy, responses, inflammation, protein, pathogenesis, disease, reveals, mycobacterium-tuberculosis, cells, immune cells, cytokines, gene, apoptosis, recovery, peripheral-blood, induction, down-regulation, b-cells, dysfunction, natural-killer-cells, transcriptomics, vaccine, package, diversity, mortality |
| 2 | Green | hpv, cancer, squamous-cell carcinoma, cervical cancer, head and neck cancer, heterogeneity, dna, survival, single-cell sequencing, landscape, rna, microenvironment, recurrent, progression, subset, machine learning, immunotherapy, hpv-16, epithelial cells, messenger-rna, p-tefb, exhaustion |
| 3 | Blue | cd4(+) t-cells, replication, reservoir, hiv latency, identification, proviruses, lymphocytes, intact hiv-1, proliferation, persistence, virus, restriction, pathway, chromatin accessibility, clonal expansion, establishment, in-vivo |
| 4 | Yellow | scrna-seq, t-cells, macrophages, dendritic cells, differentiation, individuals, receptor, blood, memory, immunity, tissue, biomarkers, antigen, spatial transcriptomics, lymphoid-tissue, regulatory t-cells |

**Table S8** Summary of clinical trials utilizing single-cell sequencing in STIs.

| NCT Number | Study Type | Study Status | Start Date | Phases | Enrollment | Single-cell technology | Study Results | Sponsor |
| --- | --- | --- | --- | --- | --- | --- | --- | --- |
| NCT07367867 | Observational | Completed | 2023 | NA | 320 | scRNA-seq | NO | Universidad de Magallanes |
| NCT06034314 | Interventional | Active, not recruiting | 2023 | Phase1 | 160 | scRNA-seq, scATAC-seq | NO | Yale University |
| NCT06016114 | Observational | Not yet recruiting | 2024 | NA | 25 | scRNA-seq | NO | University Hospital, Ghent |
| NCT05783388 | Observational | Withdrawn | 2023 | NA | 0 | scRNA-seq | NO | University Hospital, Ghent |
| NCT05668026 | Interventional | Recruiting | 2024 | Phase1\|Phase2 | 18 | scRNA-seq | NO | University of Aarhus |
| NCT05209867 | Interventional | Completed | 2022 | Phase4 | 4 | scRNA-seq | YES | University of Florida |
| NCT04920539 | Interventional | Completed | 2022 | Phase1 | 56 | single cell epigenomic and transcriptomic | NO | Yale University |
| NCT04305665 | Observational | Unknown | 2020 | NA | 25 | scRNA-seq | NO | University Hospital, Ghent |
| NCT02641756 | Interventional | Completed | 2016 | NA | 12 | single proviral sequencing | NO | University Hospital, Ghent |

The data for Figure 2 was directly exported from Web of Science, as shown in the table below, and can be visualized using a bar chart in Excel.

**Table S9** The data for Figure 2

|  | Publications | Citations |
| --- | --- | --- |
| 2015 | 1 | 0 |
| 2016 | 0 | 3 |
| 2017 | 2 | 5 |
| 2018 | 9 | 29 |
| 2019 | 4 | 74 |
| 2020 | 14 | 184 |
| 2021 | 14 | 283 |
| 2022 | 25 | 478 |
| 2023 | 31 | 549 |
| 2024 | 36 | 659 |
| 2025 | 12 | 306 |


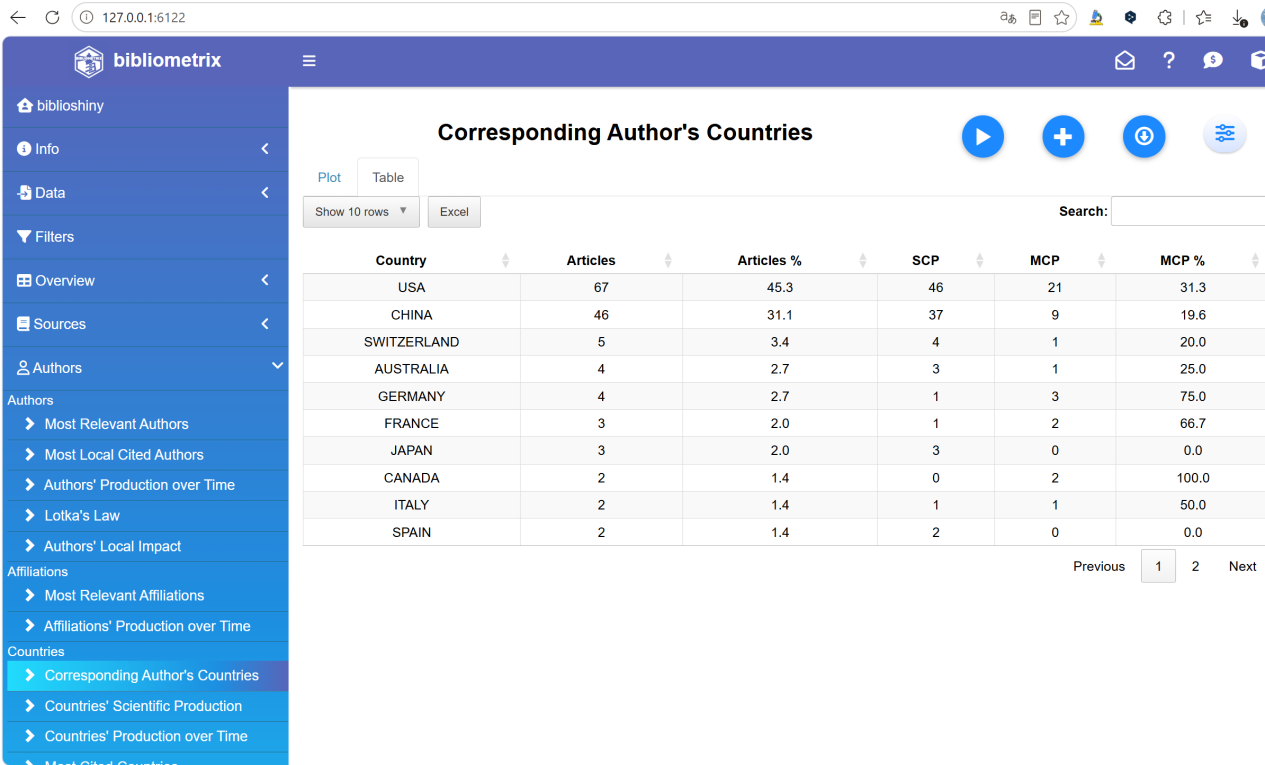


**Figure S2** The data for Figure 3A.


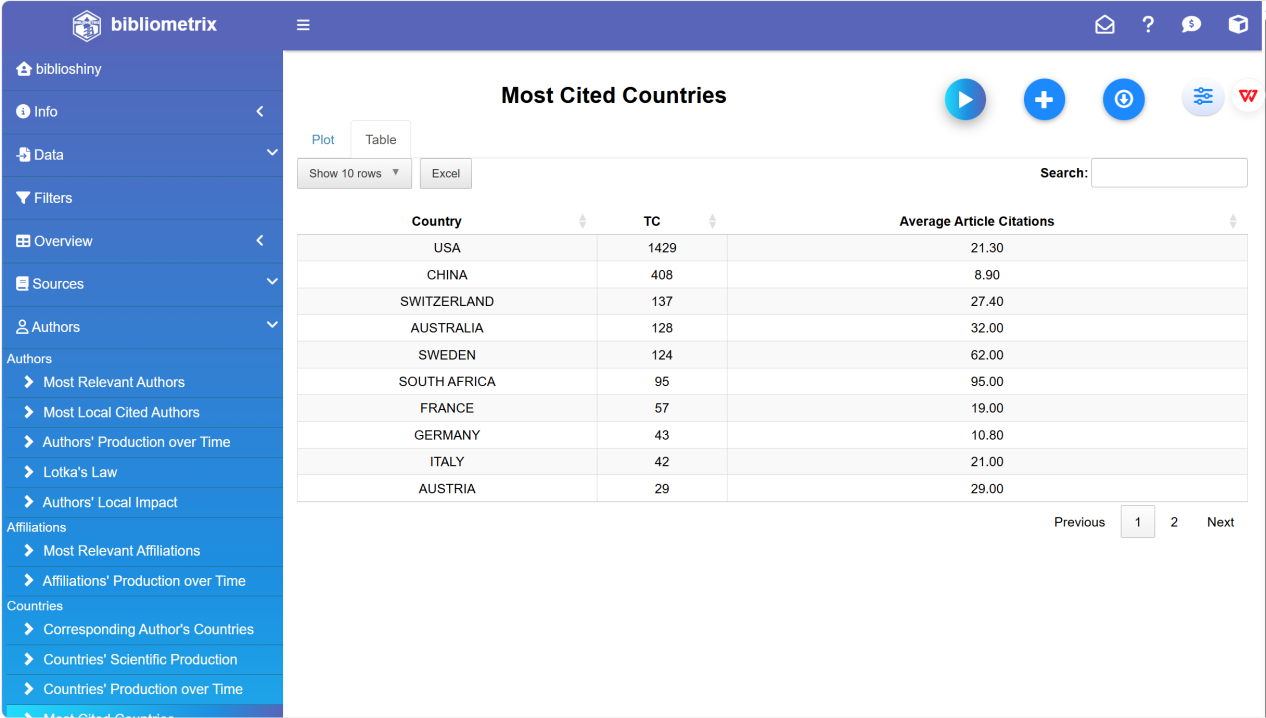


**Figure S3** The data for Figure 3B.


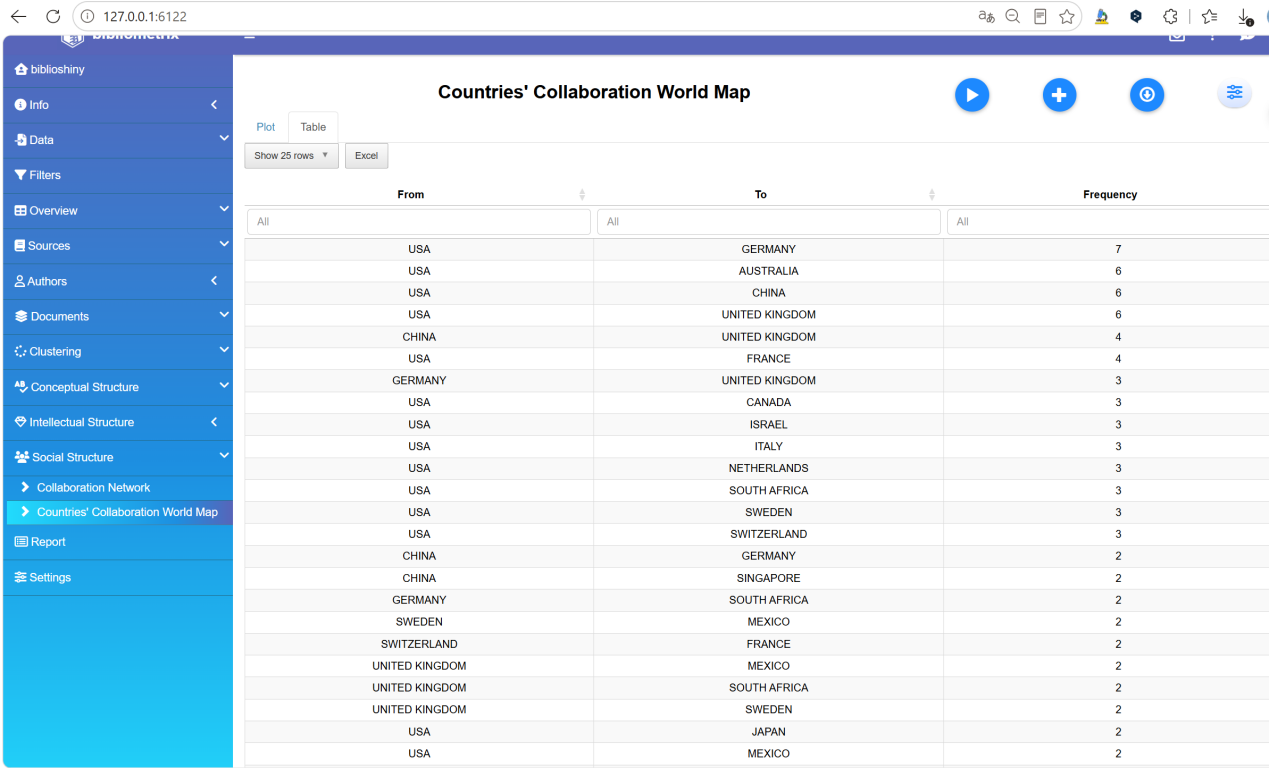


**Figure S4** The data for Figure 3C.


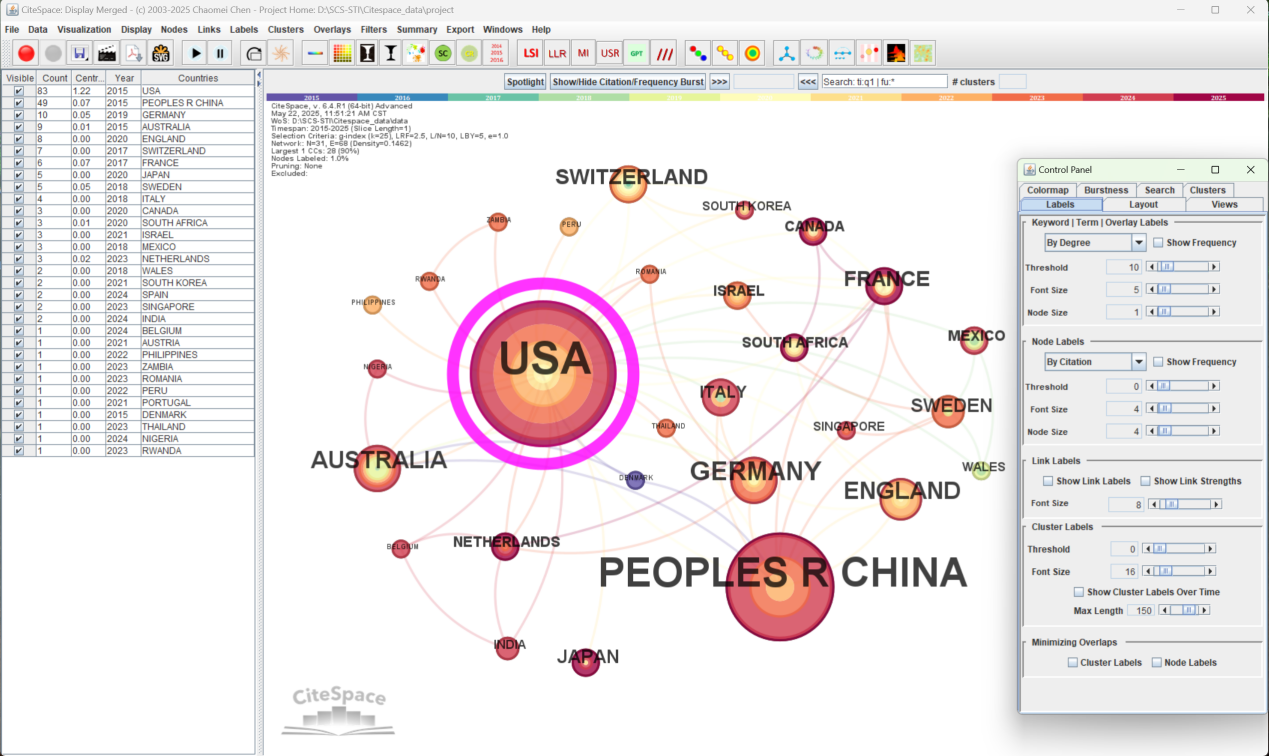


**Figure S5** Overall parameters for Figure 3D.


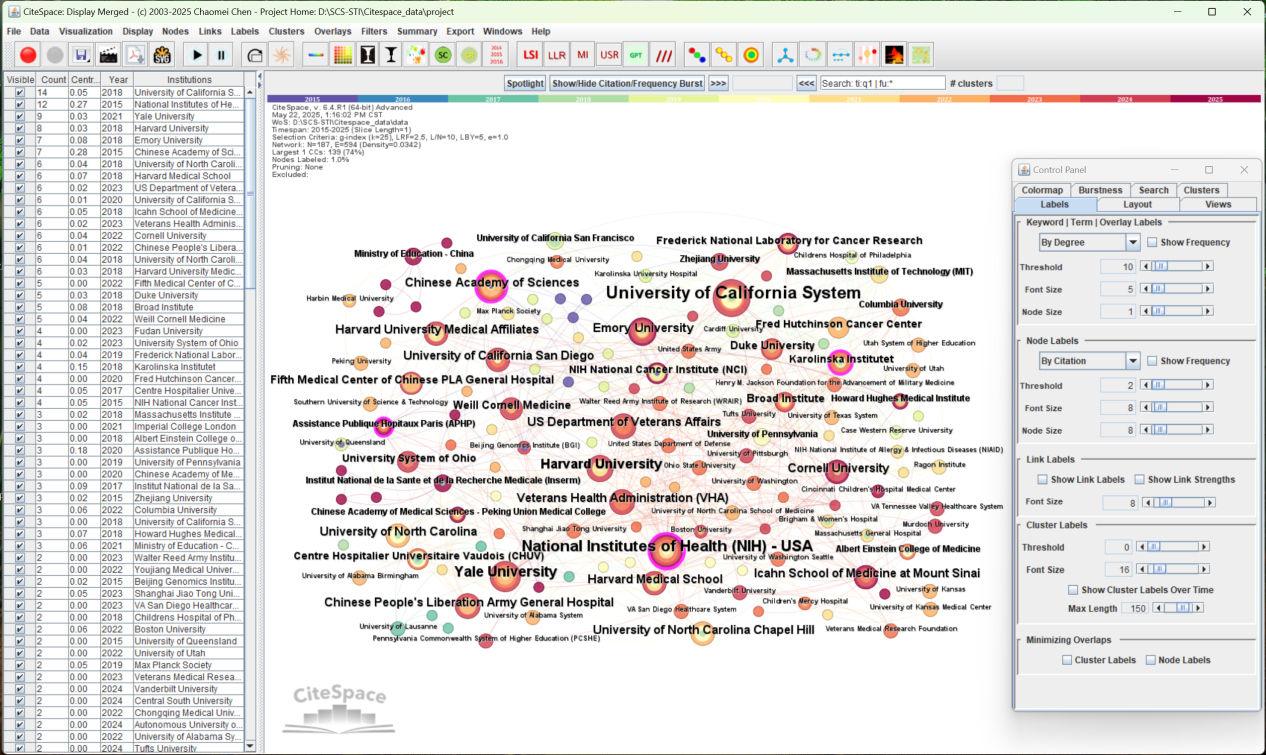


**Figure S6** Overall parameters for Figure 4A.


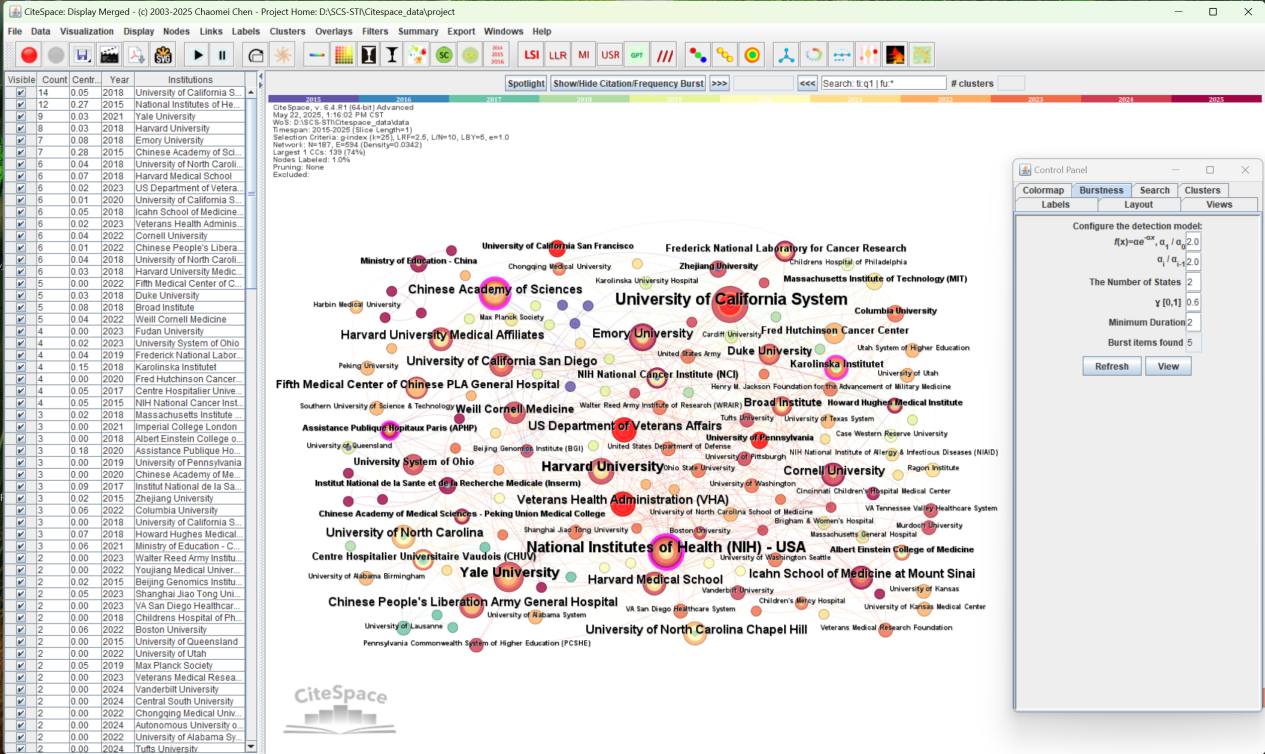


**Figure S7** Overall parameters for Figure 4B.


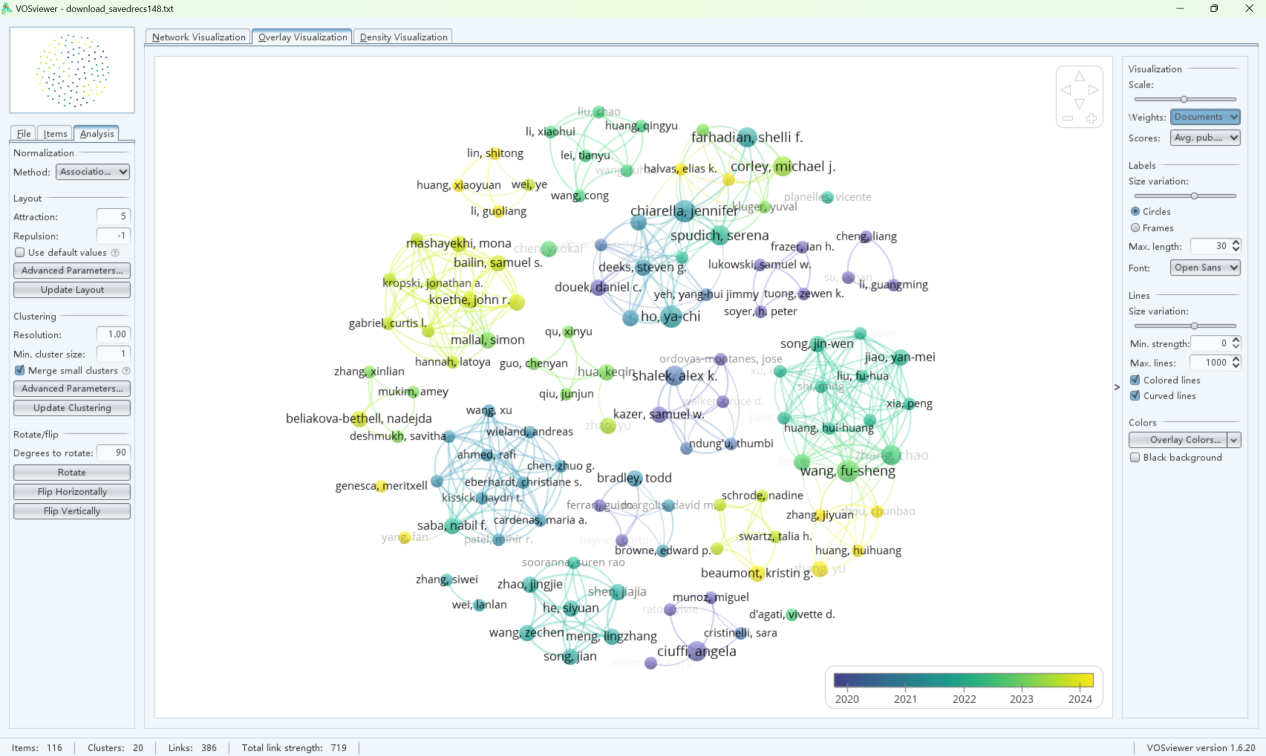


**Figure S8** Overall parameters for Figure 5A.


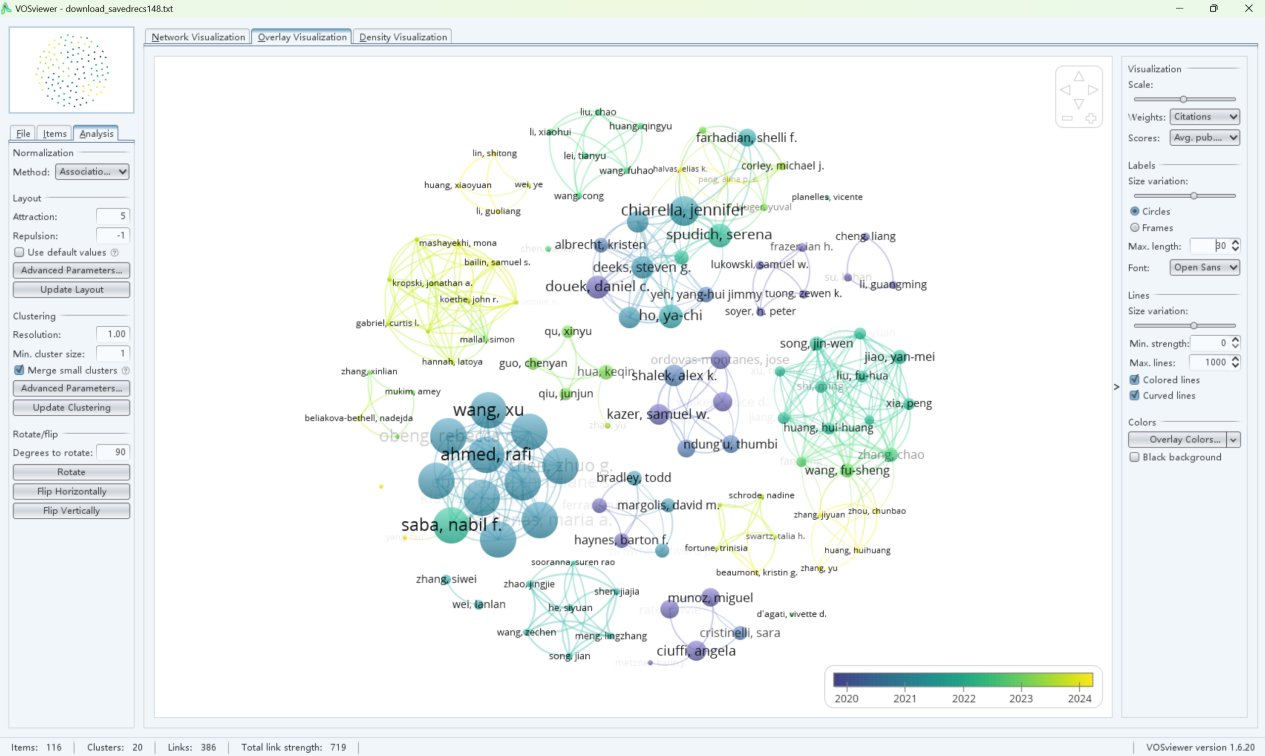


**Figure S9** Overall parameters for Figure 5B.


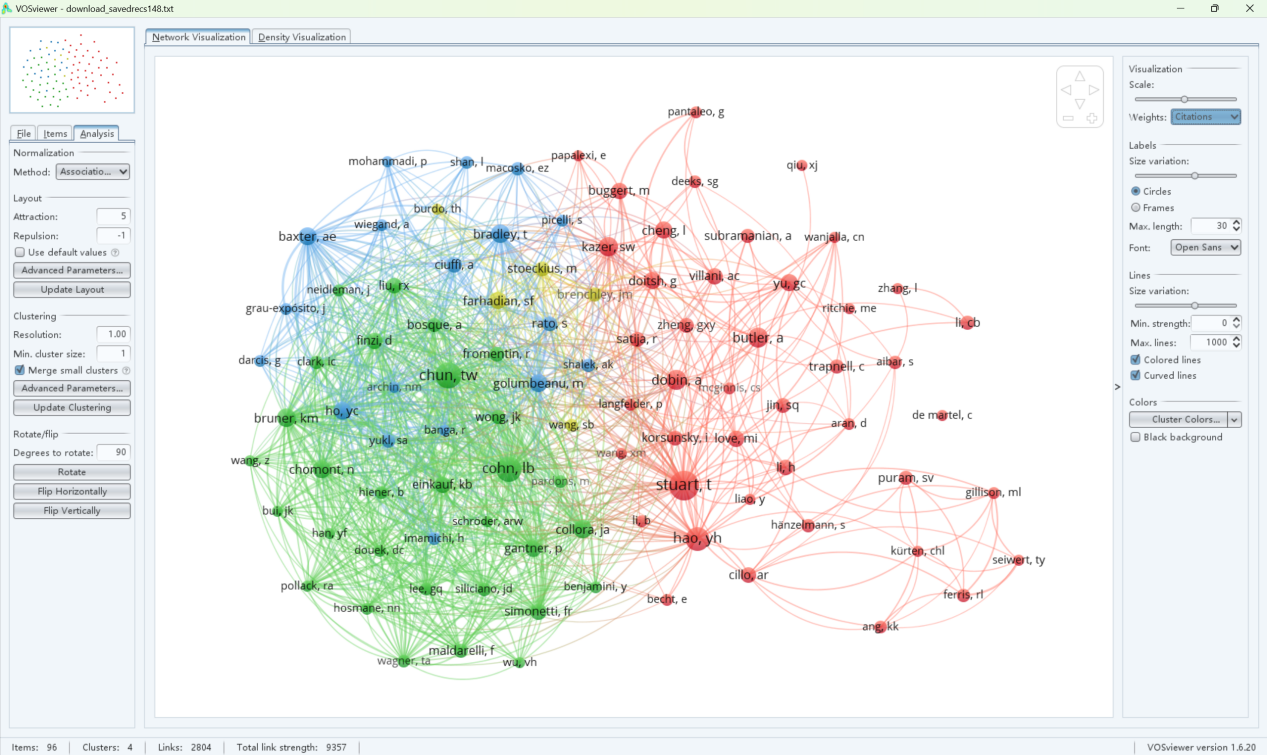


**Figure S10** Overall parameters for Figure 5C.


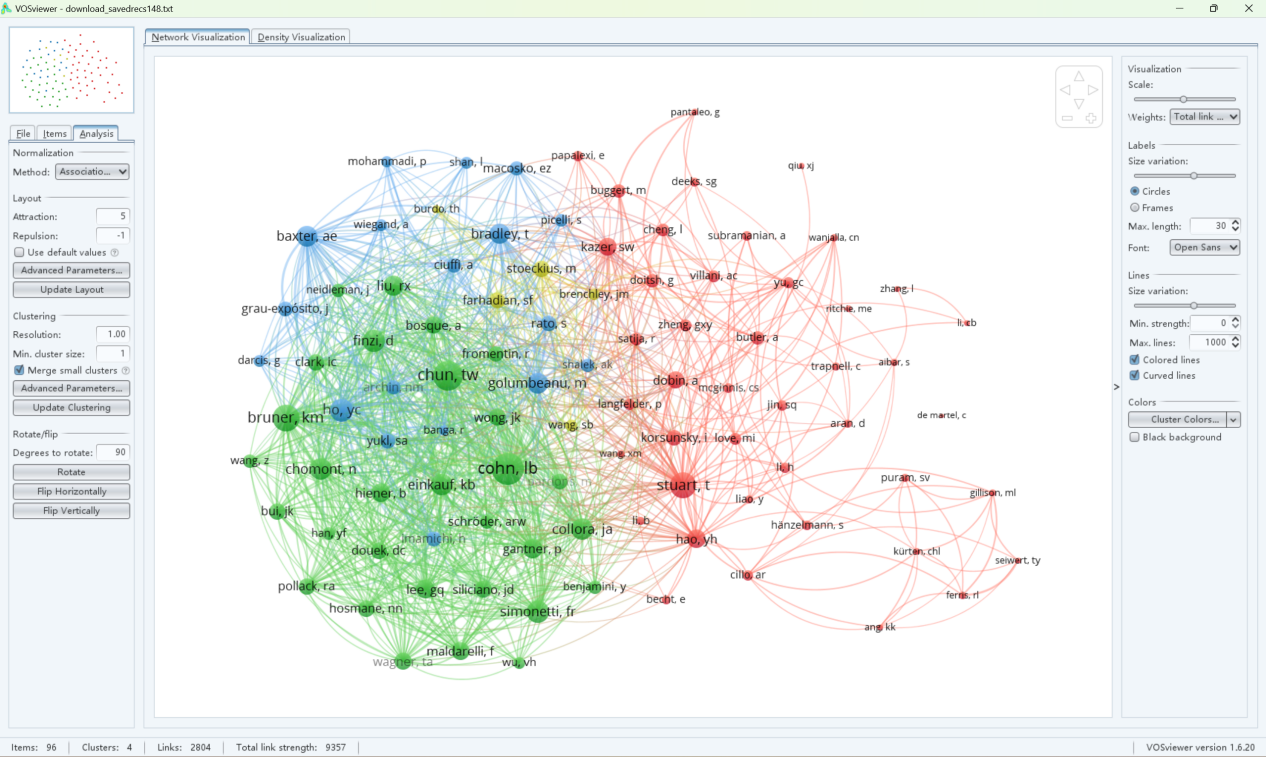


**Figure S11** Overall parameters for Figure 5D.


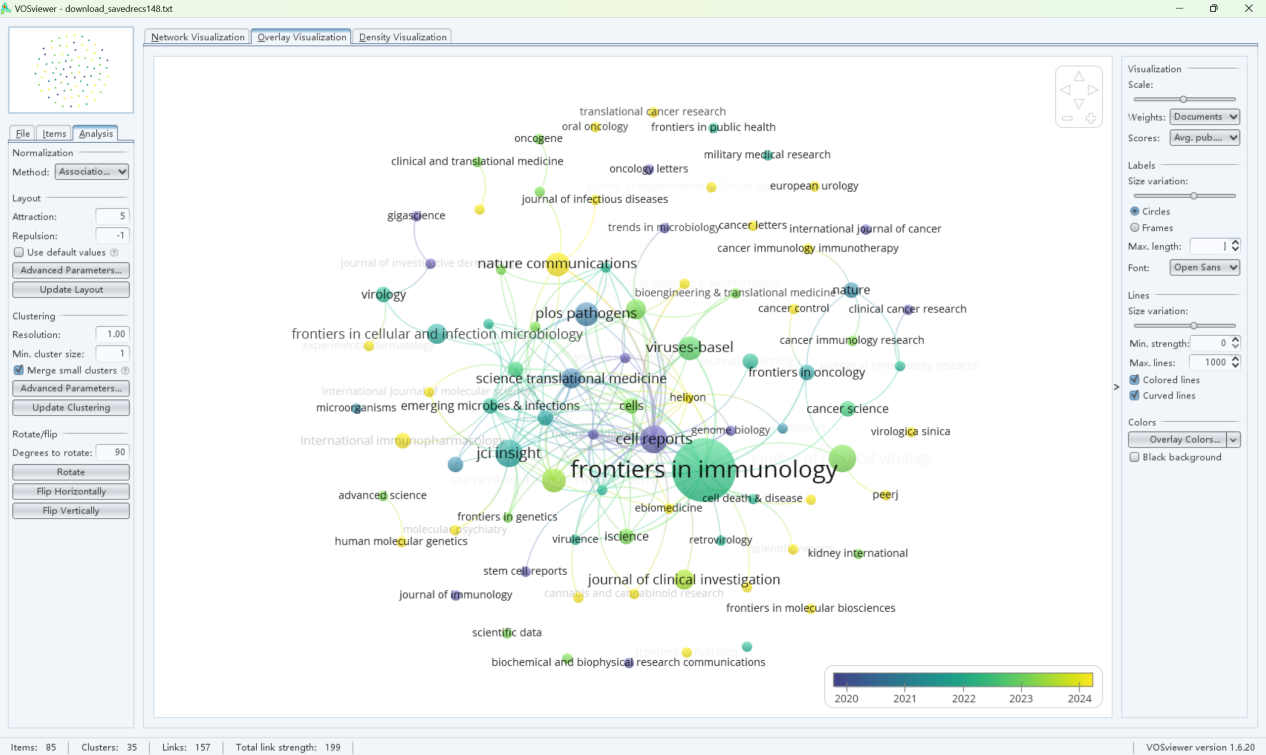


**Figure S12** Overall parameters for Figure 6A.


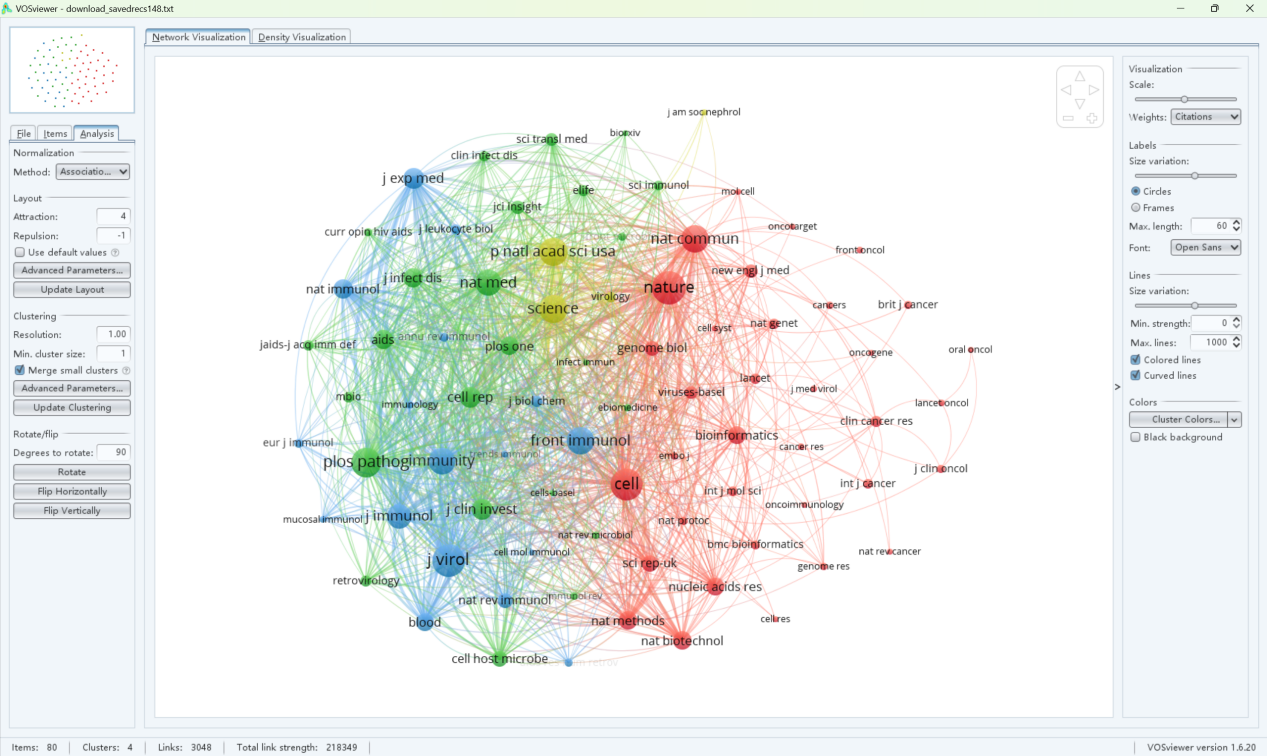


**Figure S13** Overall parameters for Figure 6B.


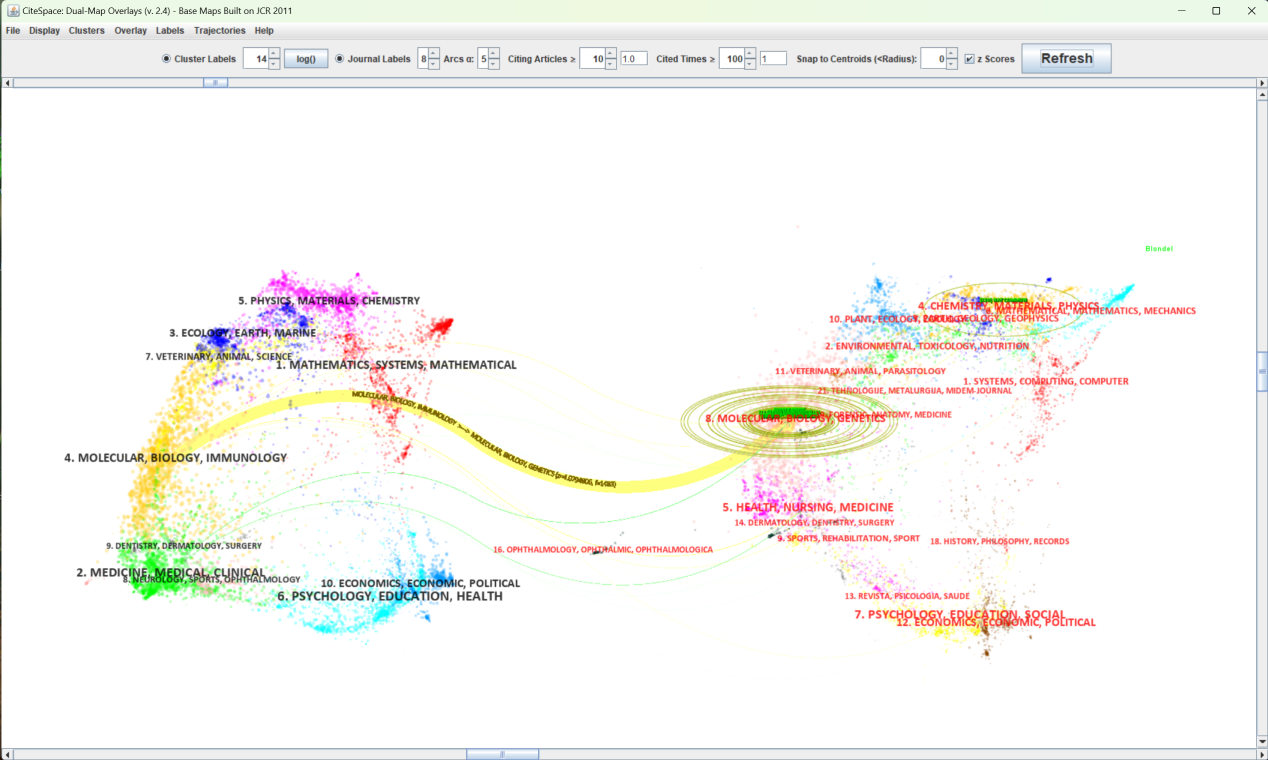


**Figure S14** Overall parameters for Figure 6C.


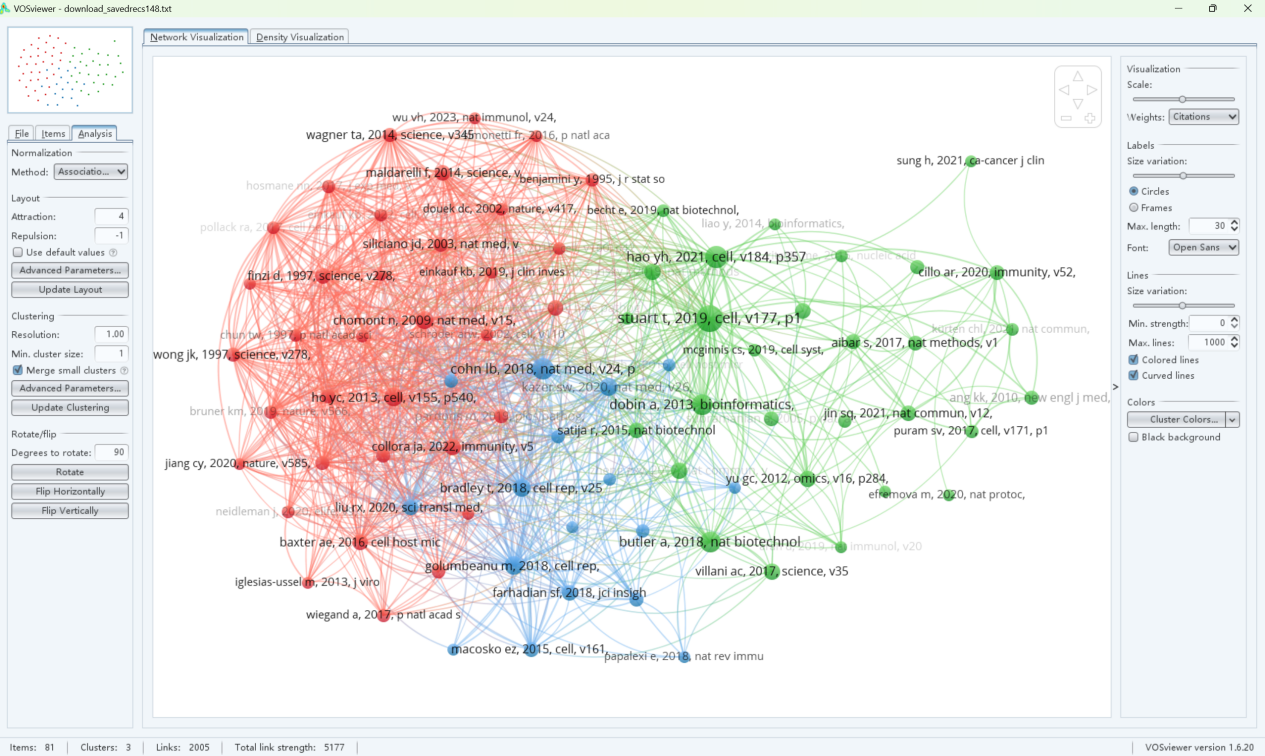


**Figure S15** Overall parameters for Figure 7A.


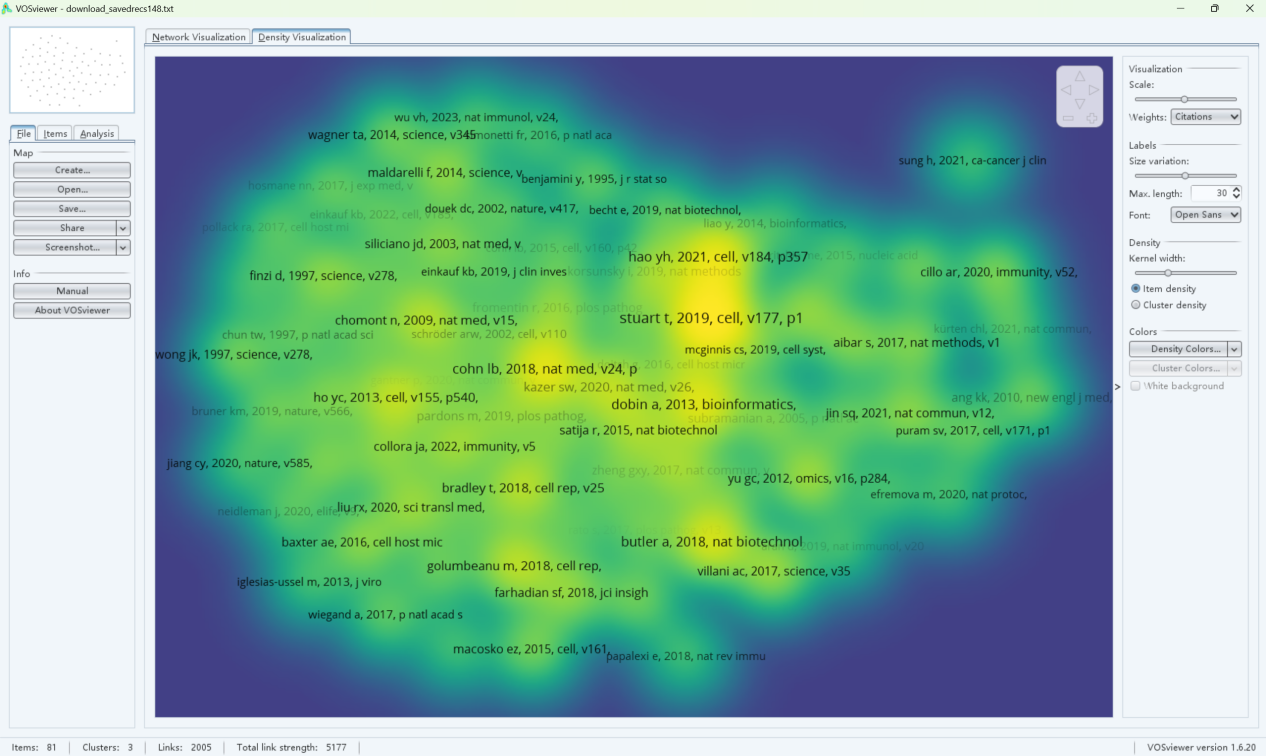


**Figure S16** Overall parameters for Figure 7B.


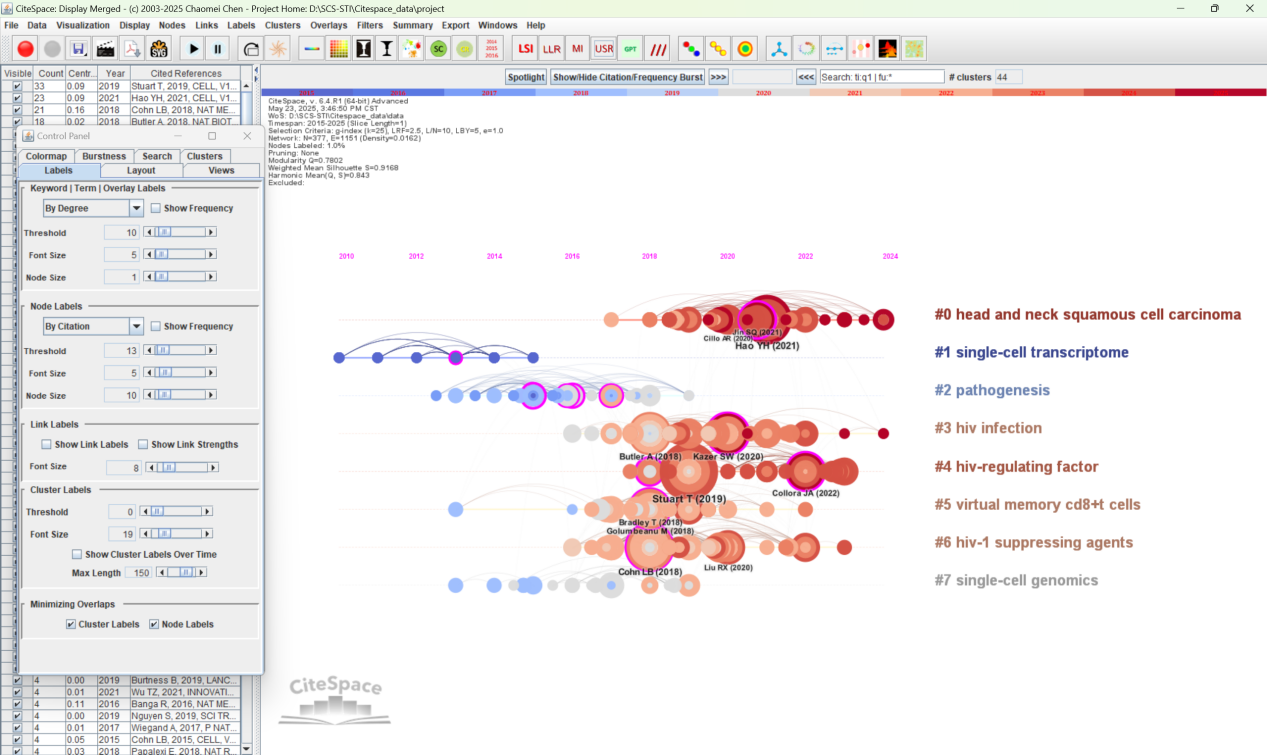


**Figure S17** Overall parameters for Figure 7C.


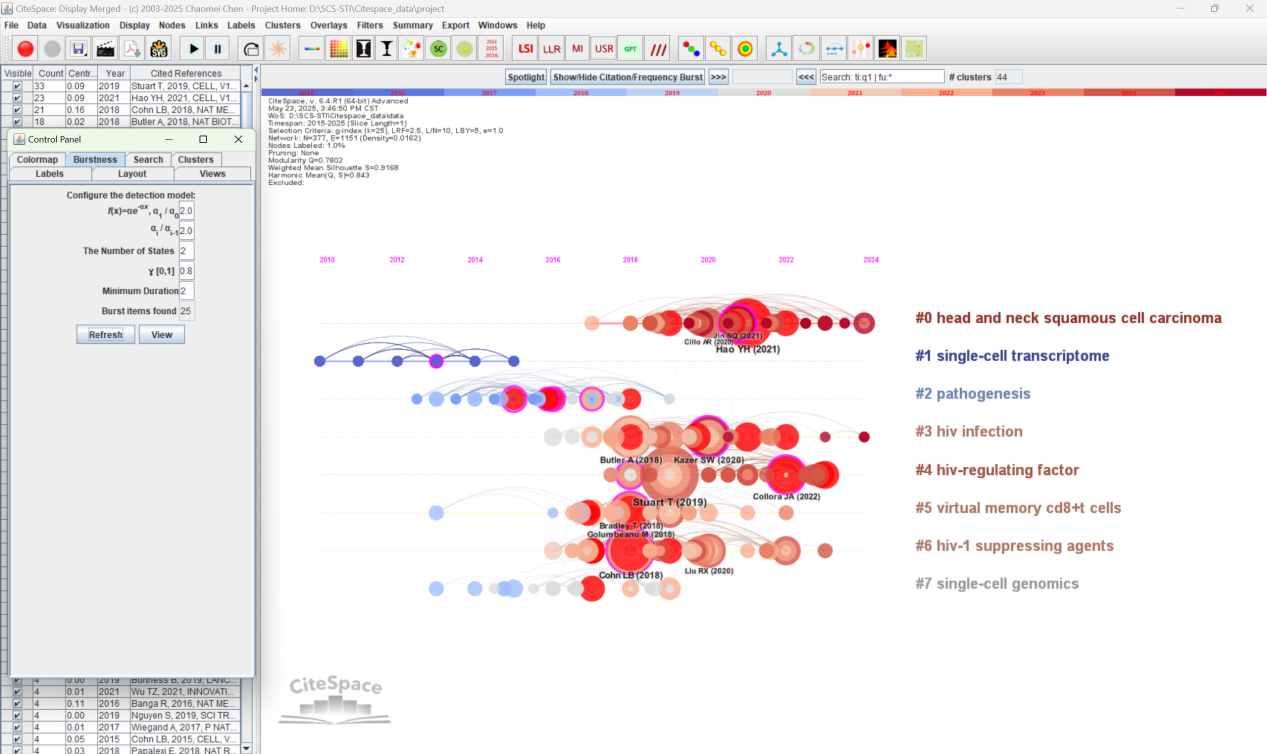


**Figure S18** Overall parameters for Figure 7D.


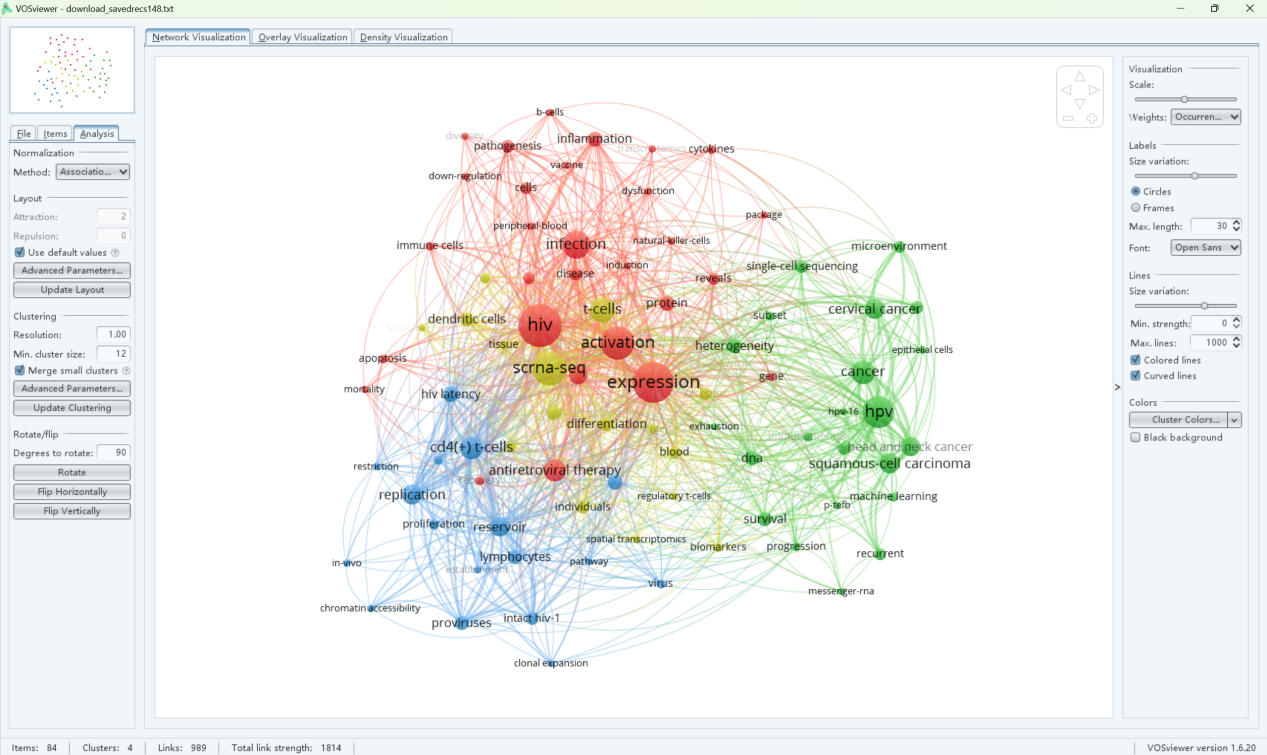


**Figure S19** Overall parameters for Figure 8A.


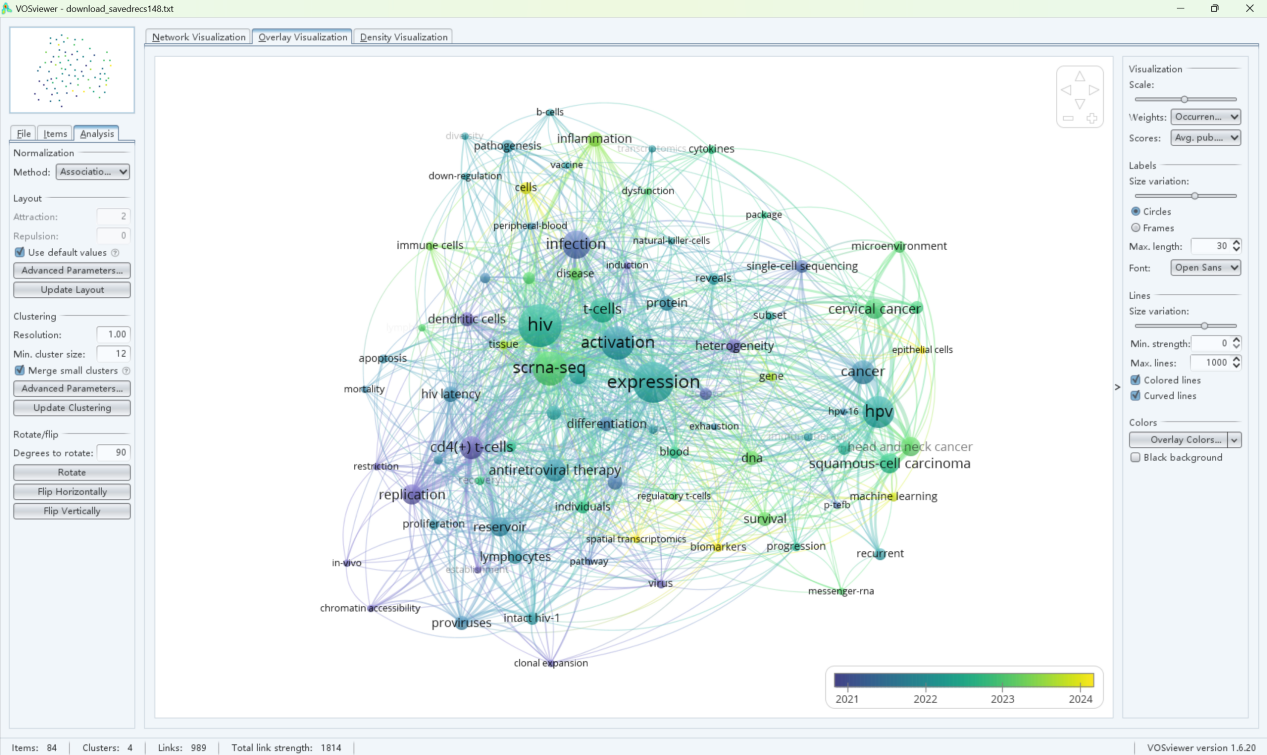


**Figure S20** Overall parameters for Figure 8B.


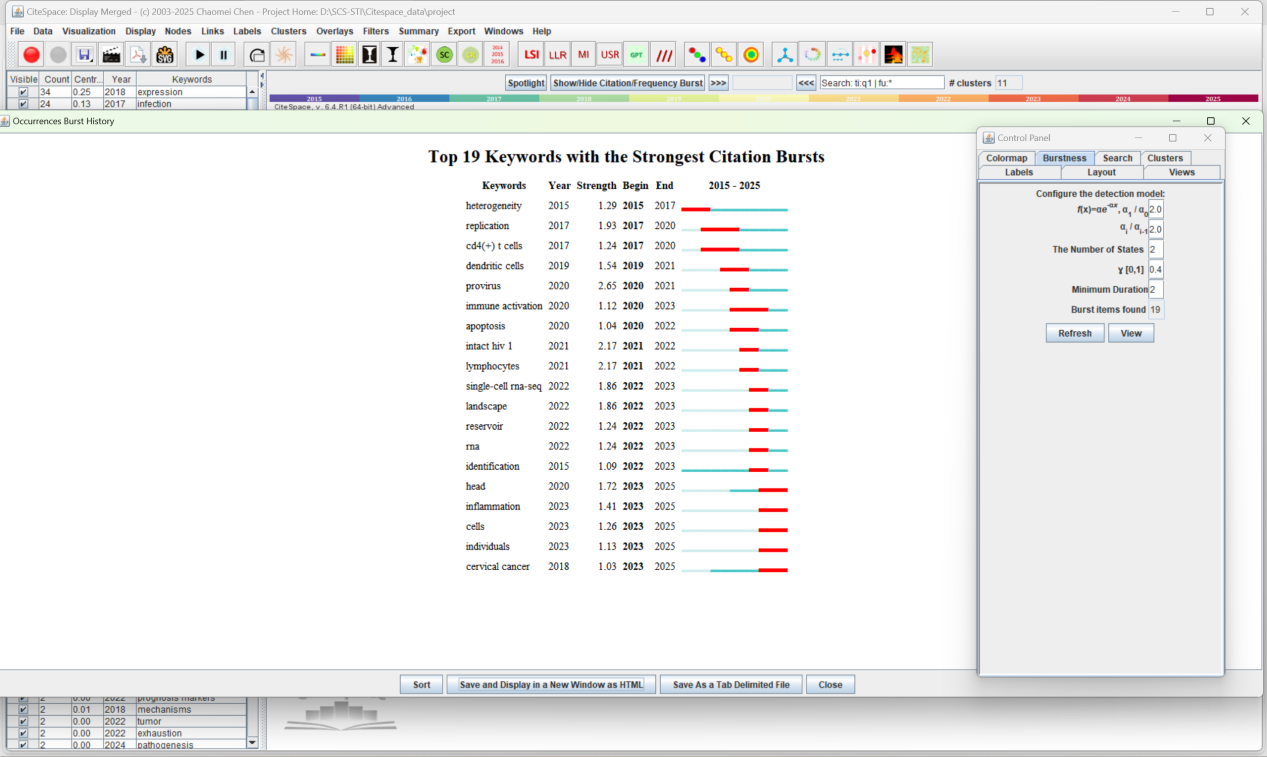


**Figure S21** Overall parameters for Figure 8C.

**Table S10** The merging of keywords with identical or similar meanings.

| keyword | replace by |
| --- | --- |
| dendritic cell | dendritic cells |
| gene-expression | expression |
| hiv-1 | hiv |
| hiv-1 infection | hiv |
| hpv infection | hpv |
| human immunodeficiency virus | hiv |
| human papillomavirus | hpv |
| human-immunodeficiency-virus | hiv |
| human-papillomavirus | hpv |
| human-papillomavirus type-16 | hpv-16 |
| immunodeficiency-virus type-1 | hiv |
| single cell rna sequencing | scrna-seq |
| single-cell rna sequencing | scrna-seq |
| single-cell rna-seq | scrna-seq |
